# ASH: A Multi‐Scale, Multi‐Theory Modeling Program

**DOI:** 10.1002/jcc.70359

**Published:** 2026-03-29

**Authors:** Ragnar Bjornsson

**Affiliations:** ^1^ University of Grenoble Alpes, CNRS, CEA, IRIG, Laboratoire de Chimie et Biologie des Métaux Grenoble France

## Abstract

We introduce ASH, a multi‐scale, multi‐theory modeling program for quantum mechanics (QM), molecular mechanics (MM), and hybrid calculations, written in the Python programming language. ASH is written in response to the increasingly diverse computational chemistry software landscape that features more QM and MM programs than ever before, and with machine learning interatomic potentials (MLIP) further changing the way modern computational chemistry is being performed. ASH is a Python library that intentionally separates computational chemistry jobs (geometry optimizations, frequency calculations, molecular dynamics, surface scans, reaction paths, etc.) from the QM, MM, or ML method (calculated by the specialized QM or MM programs or ML libraries). By keeping the jobs separate from the Hamiltonian, a highly flexible computational chemistry environment emerges that can be used in workflows involving QM methods (using interfaces to many different QM programs), classical MM methods with multiple force fields (via an interface to the OpenMM library), machine‐learning potentials, or hybrid methods. ASH is especially powerful as a program for performing hybrid simulations: including QM/MM, QM/ML, ML/MM, QM + ML, or ONIOM calculations for proteins, solvated molecules, or molecular crystals. Molecular dynamics and enhanced sampling can be performed using any level of theory allowing for highly flexible free‐energy simulations (such as metadynamics) enabled by interfaces to algorithms in OpenMM and Plumed. There are flexible interfaces to many QM programs such as ORCA, xTB, pySCF, CP2K, MRCC, Turbomole, CFour, and many others.

## Graphical abstract

ASH is a Python‐based computational chemistry software for multi‐scale and multi‐theory computations (including QM/MM and ONIOM) and MD simulations. The program is designed for flexible workflows of molecular and biomolecular systems, allowing geometry optimizations, numerical frequencies, high‐level thermochemistry, MD and free‐energy simulations, NEB reaction paths, and much more at any level of theory. Interfaces to many external QM programs as well as ML potentials are available.

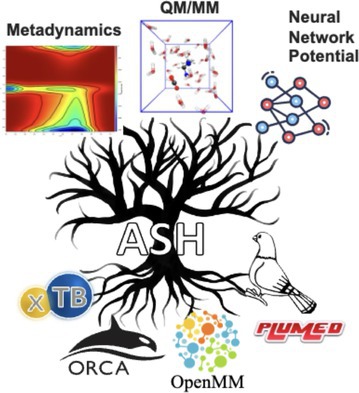

## 1 Introduction

The computational chemistry software landscape has undergone a radical change in recent years as it is no longer dominated by a few general‐purpose quantum chemistry codes (or similarly a few general classical molecular mechanics codes) as was the case only a few years ago. Instead, quantum chemistry software is seeing (i) increased specialization, for example, new codes dedicated to specialized methodology such as semi‐empirical methods, density functional methods, correlated wavefunction methods, quantum Monte Carlo, and so forth (ii) new linear scaling quantum mechanics (QM) codes exploring new developments in basis set treatment, and (iii) codes exploiting modern hardware developments, for example, GPUs and other processing units. There is furthermore increased diversity w.r.t. the licensing model: commercial codes, free‐for‐academic‐use closed‐source codes, and open‐source codes. Modern trends in open‐source software and package ecosystems have further affected scientific software distribution, and nowadays it is common to see computational chemistry methods and algorithms in various forms of implementations on source‐code repository hosts such as GitHub and GitLab.

It is clear that the field of computational chemistry software has completely changed, and it seems unlikely for it to go back to the previous dominance of a handful of programs. However, these developments come with drawbacks. Developers of specialized QM programs implementing a novel interesting method may struggle to attract users for their method unless the program has implemented the typical job types and algorithms that the user expects to be present in a general QM code. We echo opinions about the growing importance of reusable open‐source libraries in computational chemistry [1] that avoid software developers spending time and effort implementing known algorithms or methods.

Even so, new developments implemented in new programs are not as likely to make their way into the hands of a computational chemistry researcher who may have primarily learnt to use one program for their work. Users of computational chemistry codes sometimes choose the software based on the availability of a specific electronic structure method or perhaps the speed of the quantum chemistry implementation, but probably also often due to the availability and reliability of specific job types, for example, efficient internal‐coordinate geometry optimizations or reaction path methods for finding transition states. In molecular computational chemistry, a typical user expects to be able to reliably perform common jobs or tasks such as geometry optimizations, vibrational frequency computations, surface scans, some kind of saddle‐point search, sometimes molecular dynamics, and often be able to use these job‐types within a hybrid QM/MM or ONIOM scheme. Each QM program typically differs in the quality of the implementations of these job types, depending on the priorities of the software project. Some programs feature robust state‐of‐the‐art quasi‐Newton optimizers with a robust choice of internal coordinates with, for example, constraints implemented, while other programs have opted for simpler easy‐to‐code Cartesian optimizations, often without constraints. Classical molecular mechanics (MM) codes, primarily intended for MD simulation similarly often feature rudimentary minimization algorithms. Yet the ingredients that go into a geometry optimization, surface scan, numerical frequencies, reaction path optimization, or MD simulation of any N‐atom system are the same: the system energy and the system 3 N gradient. Many of these “job type” algorithms are actually most of the time completely independent of the Hamiltonian (whether QM or MM).

Separating out these “job types” (that only depend on energy and gradients of some coordinates) of computational chemistry into another program or library allows for much more flexibility. Having a driver program responsible for carrying out a geometry optimization or molecular dynamics that communicates with QM or MM programs by energy and gradient information alone allows one to easily perform geometry optimizations or Born‐Oppenheimer molecular dynamics at any level of theory using any QM or MM program that features an interface.

Furthermore, such a driver program with interfaces to different QM or MM programs allows a general common QM/MM scheme where a QM‐method Hamiltonian in any program could be combined with any MM method Hamiltonian. Additionally, having a single program with interfaces to multiple QM programs that have specialization in different areas (molecular DFT, periodic DFT, semi‐empirical tight binding, coupled cluster, multi‐reference methods, quantum Monte Carlo) may allow many unexplored opportunities for hybrid theories. Combining the results of multiple quantum chemistry programs can allow for multi‐level, multi‐program workflows, where energy and even gradients are combined from different sources in various ways. High‐accuracy correlated WFT composite methods where a total energy is estimated as the sum of contributions from HF, MP2, CCSD(T), and sometimes post‐CCSD(T) (the latter only available in a handful of QM programs) are an obvious example, while low‐level→high‐level Δ‐machine‐learning simulations are a modern, less obvious example.

ASH is written in response to these current developments in the field of computational chemistry that have led to a zoo of quantum chemistry programs and the advantages of separating job types from electronic structure methods. This approach to computational chemistry is not novel as it has caught on in the solid‐state chemistry and physics community, where the Atomic Simulation Environment (ASE) [2] has become a popular Python library implementing various tools to setup solid‐state systems, implementation of various minimization and simulation algorithms, reaction path algorithms, as well as interfaces to many DFT programs. This approach is less common in the molecular or biomolecular chemistry community. The Chemshell program [3, 4] may have been one of the first examples of this type of computational chemistry software but the program never caught on more broadly, probably in part due to the licensing model but also due the less popular scripting language TCl. A rewrite of Chemshell with a Python frontend is now available [5]. Exploration of ground and excited energy surfaces in a package independently of what individual QM programs offer is the subject of the pysisyphus [6] package. Other interesting projects with some shared philosophy to ASH include, for example, pDynamo [7, 8], Cuby [9], QMCube [10], QCEngine [11], Cclib2 [12], PyDFT‐QMMM [13].

In this article, we describe ASH, primarily from a potential user's perspective. It is a Python library that separates computational chemistry jobs (e.g., geometry optimization, frequency calculation, molecular dynamics, surface scans, reaction paths) from the Hamiltonian (calculated by the specialized QM or MM programs). By keeping the jobs separate from the Hamiltonian, a common interface is possible that gives the user access to both old and new electronic structure methods while enabling both standard and specialized calculations and workflows. ASH is also a general program for hybrid methods, allowing for general QM/MM and ONIOM coupling, differing from specialized QM/MM implementations that only combine a single QM program with a single MM program.

## 2 General Description of ASH

**FIGURE 1:**
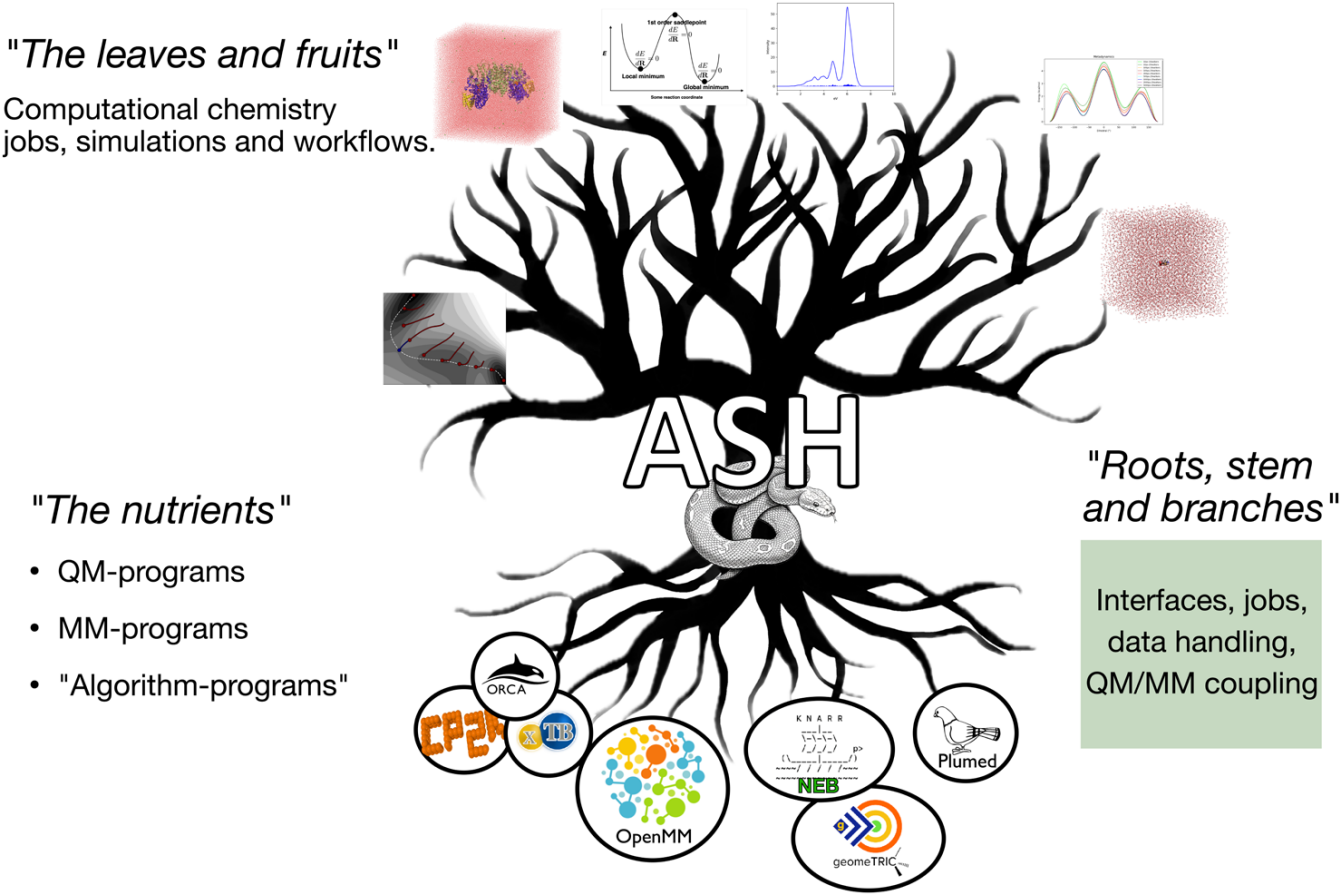
ASH can be thought of as the roots, stems, and branches of a tree (interfaces, data handling, hybrid QM/MM coupling, etc.), enabling “nutrients” (the output of QM, MM, and ML potentials) to be combined and converted into “leaves and fruits” (advanced simulations and workflows).

The name ASH is actually not an acronym, but refers to the ash tree of Yggdrasill, the world tree in Norse mythology. The logo, shown in Figure 1, acts as a convenient metaphor where the program can be thought of as the roots, stems, and branches of an ash tree turning nutrients (energies, gradients, and wavefunctions from various programs) into leaves and fruits (computational workflows and simulations). ASH is hence a Python library that deliberately separates the problem of computing complex electronic structure or classical potentials from the computational chemistry jobs and simulations the user seeks to perform. Instead, ASH provides convenient interfaces to a plethora of QM programs, a flexible state‐of‐the‐art MM and MD library (OpenMM [14]) as well as programs featuring minimization or dynamics algorithms, enabling advanced simulations to be carried out in a flexible way.

In the conception of ASH, we decided early on that the program needed to be easy to install, requiring no compilation and for that reason we have deliberately stayed away from complex interfaces between high‐ and low‐level programming languages. Instead, ASH is mostly pure Python while relying on the NUMPY library [15] (written in C) for efficient numerical computations when needed. Some specialized functionality relies on an interface to the high‐performance just‐in‐time compiled Julia language. ASH is easily downloaded and imported as a Python module in any Python environment or can be installed by a package manager (currently pip). One uses the program by writing a Python script (or possibly a Jupyter notebook), and imports specific functionality from the ASH library. This approach, as opposed to a bespoke input file syntax, has the advantages of exploiting the strengths of Python as a general easy‐to‐learn scripting language, giving the user power to use ASH in a flexible way and with a familiar scripting language syntax. The popularity of Python in data science and data visualization also allows for easily combining ASH functionality within already established workflows.

The ASH functionality is present in the form of classes and functions available to the user, once importing the ASH library. In a typical setting, the user writes a simple Python script and either runs it directly by the Python interpreter or submits as a job via a scheduler (a submission script intended for the Slurm job scheduler [16] is available to users). While ASH internally makes use of Python classes and object‐orientation (OO), we have deliberately chosen to avoid exposing the user to OO concepts (e.g., in the form of calling a method within an object). Instead, we have preferred the syntax where the user creates objects corresponding to a molecular system (of the **Fragment** class) and a theory‐level (of a **Theory**‐type class) but then runs a computational chemistry job by calling a standalone function, taking the **Fragment** and **Theory** objects as input. This creates, in our opinion, an easier‐to‐use and easier‐to‐learn environment, especially for users less familiar with Python or scripting/programming.

**FIGURE 2:**
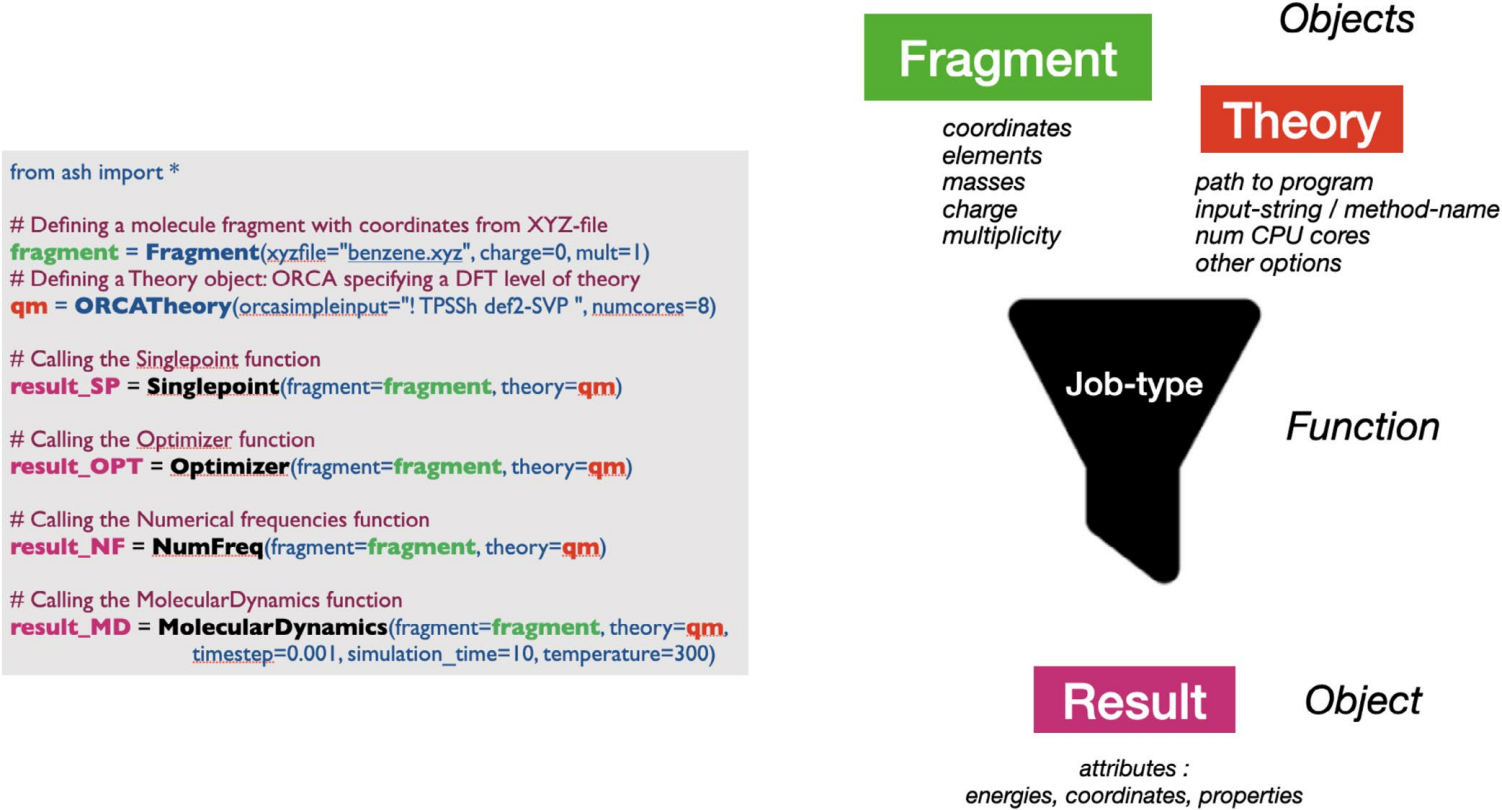
A basic ASH script showing how to create a Fragment (here reading coordinates of benzene from an XYZ‐file), and a **Theory** object (here using the ORCA interface and specifying a TPSSh/def2‐SVP Hamiltonian), and how single‐point energy, geometry optimization, numerical frequency, and molecular dynamics jobs can be run using the **Fragment** and **Theory** objects as input.

A **Fragment** object serves the purpose of representing the coordinates of the molecular system within ASH, and can be created in many different ways from Cartesian coordinates (e.g., from an XYZ‐file, PDB‐file, Python lists, NUMPY arrays or a multiline‐string). The object additionally stores element information, masses, charge, spin multiplicity and sometimes system topology and connectivity (when needed). A **Theory** object, on the other hand, represents both the theory‐level and external‐program interface, that will be applied to calculate the state and energy of the system. The object will contain information about how the external program should run, how parallelization is handled, path to the external program or possibly a direct link to the Python API. A **Theory** object is usually created from one of the many available interfaces in ASH or it may be a hybrid theory. A hybrid **Theory** object combines two or more **Theory** objects for the purpose of performing, for example, QM/MM or ONIOM coupling. Once **Fragment** and **Theory** objects are available, a user can call a job‐function corresponding to the type of job to be carried out. Typical job‐types include *Singlepoint*, *Optimizer*, *NumFreq*, *MolecularDynamics*, and *Surface_scan*, all taking one **Fragment** object and one **Theory** object as input. The output of most job‐functions is a **Results** object (a Python Dataclass) that will contain data such as energy, geometry, vibrational frequencies, and so forth, depending on the job‐function that was called. Figure 2 shows an example of a basic ASH script.

**FIGURE 3:**
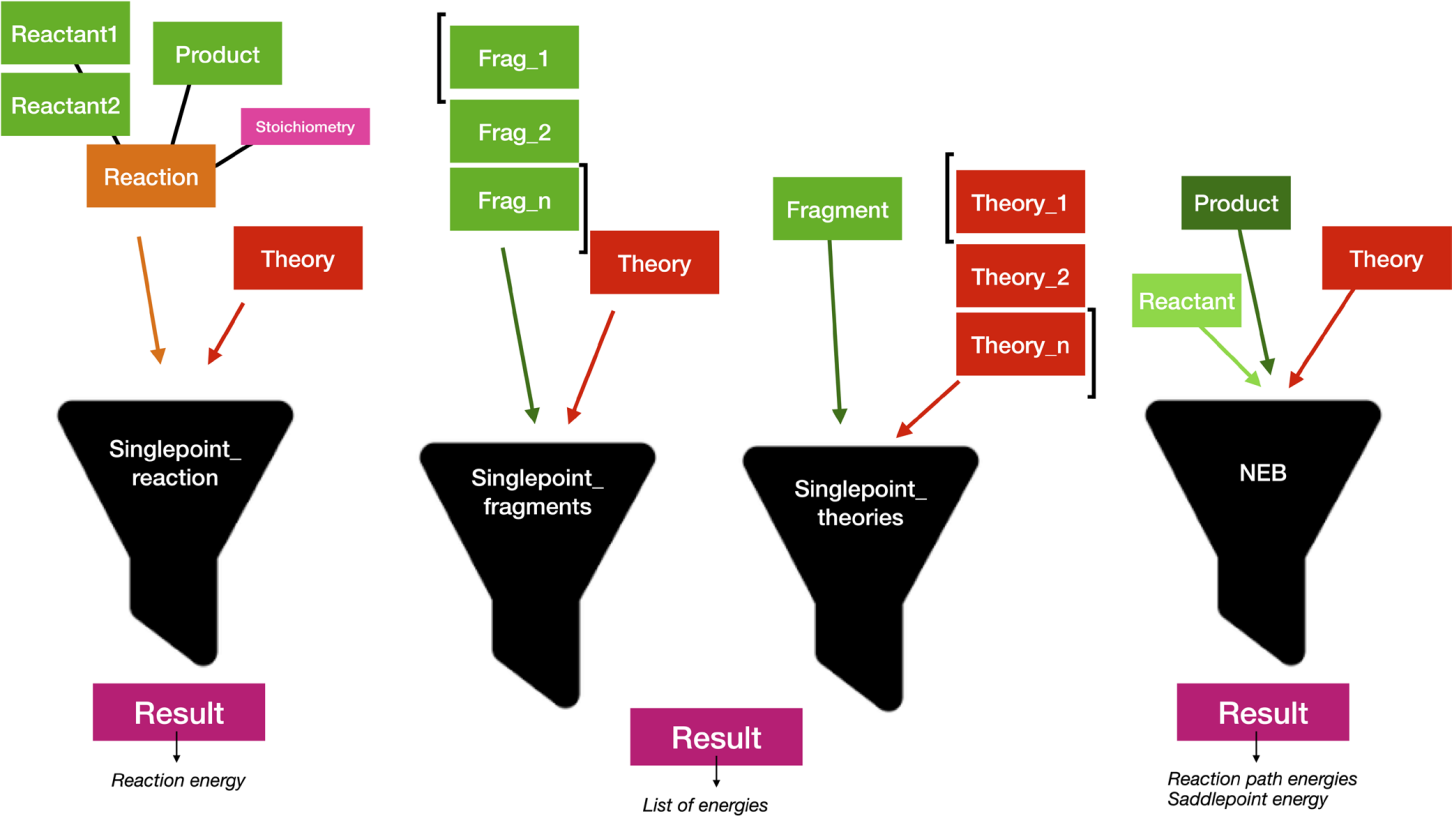
Examples of ASH job functions taking lists of multiple **Fragment** and/or **Theory** objects as input for multi‐step workflows.

Jobs in ASH are almost exclusively standalone functions, rather than methods associated with the classes (internally, the classes contain methods, typically not intended to be called by the user). Some job‐types may take more than one **Fragment** object or **Theory** object as input. As an example, the NEB function takes two **Fragment** objects as input (corresponding to a reactant geometry and a product geometry) to allow interpolation and minimization of images along a reaction path between reactant and product. There are also job‐types that take multiple Fragments or multiple **Theory** objects as input instead, for the purpose of carrying out multistep workflows as shown in Figure 3.

## 3 Interfaces to QM and MM Programs

Without interfaces to useful QM or MM programs, ASH would not be capable of much. We have chosen to write interfaces to what we consider among the most useful molecular QM programs, with emphasis on programs that either have a good selection of general electronic structure methods with robust and fast implementations (e.g., ORCA [17, 18], MRCC [19], and pySCF [20, 21]) or programs with specialized methods (e.g., xTB [22] featuring extended tight‐binding methods [23, 24], or CFour [25] for coupled cluster methods). An important factor in choosing QM‐program (to use or write an interface for) is also that they are easy to install and programs that deploy downloadable binaries that avoid compilations (e.g., ORCA, MRCC) or distribute via package managers such as pip or conda (e.g., pySCF, OpenMM, xTB, CP2K [26], and NWChem [27]) offer considerable benefits when combined with ASH. However, ASH also features interfaces to programs requiring manual compilation, for example, CFour due to the unique coupled‐cluster (CC) functionality (analytical derivatives of many CC approximations for example). Additionally, we have begun to support QM codes that have been written for GPU execution such as TeraChem [28], QUICK [29], and gpu4pySCF [30, 31] that are beneficial for exploiting GPUs for quantum chemistry.

Some of the external programs are Python libraries (or feature a Python API) and ASH will automatically detect (when the interface is called) if the library is available and warn the user with instructions on how to install. Similarly, ASH will by default detect the presence of external executable programs (e.g., ORCA) by searching the shell PATH variable (when the interface is called) and warns the user if the program executable cannot be found. Thus, ASH does not have a strict dependency list, rather the installation of external programs depends entirely on how the user wants to use ASH. We have tried our best at making sure that “program‐not‐found” error messages upon attempting to use an interface points the user in the right direction about how to enable the interface.

Parallelization of QM‐codes is an important aspect of their usage and the codes differ depending on whether they use MPI, shared‐memory parallelization (such as OpenMP), or both. Each interface in ASH will by default assume 1 CPU core which can be changed by the *numcores* keyword variable, a keyword common to all interfaces. Depending on the specific interface in question, the *numcores* variable is then passed onto the QM‐program in a specific way. In the case of MPI‐parallelization the interface will first check for the availability of the MPI library (usually OpenMPI). In the case of ORCA, the *numcores* information is written to the input file (as ORCA calls OpenMPI on its own) while other programs are executed by a mpirun‐np *numcores* programname line. For OpenMP parallelization, the OMP_NUM_THREADS environment variable is set to the *numcores* variable. A few programs can utilize a mixed MPI/OpenMP strategy (e.g., CP2K, Block2), in which case more information needs to be provided by the user.

In developing interfaces to all the different QM programs, we have followed a strategy of having the interface be as flexible as possible, allowing the user to use almost any method implemented in the QM‐program. Hence, we have for the most part tried to avoid pre‐programmed methods and basis keywords and instead left it up to the user to define the Hamiltonian in the program via either an input‐string or input dictionary. This requires the user to have some knowledge of the features of the respective QM‐program (although we also provide clear examples in the documentation on basic use of each interface). This also has the consequence that the different interfaces have required different styles in their writing, while making sure that the interfaces overall remain compatible with general ASH functionality.

While ASH features interfaces to many programs, not all programs can obviously be supported. It is relatively straightforward, however, for a new user to create a new interface to a program. The process is easy to accomplish as long as the program in question features a simple way of accessing the energy, gradient, Hessian, and so forth from disk or a Python API. The online documentation of ASH features a guide to creating new interfaces. In the next section, we describe a few of the currently available QM‐program interfaces in ASH.

### 3.1 Interface to ORCA

ORCA is molecular chemistry code dedicated to diverse types of electronic structure methods, including density functional theory, semi‐empirical theory, and both single‐reference and multireference wavefunction theory. It features state‐of‐the‐art approximations to Coulomb, Exchange, and correlation integrals which enable fast pure‐DFT, hybrid‐DFT, and MP2 calculations. In addition to standard algorithms for single‐reference WF methods (coupled cluster (CC), MP2, MP3) and multireference WF methods (CASSCF, NEVPT2, MRCI), ORCA features pioneering domain‐based local pair‐natural‐orbital based approaches (DLPNO [32, 33, 34, 35]) that avoid the prohibitive scaling of, for example, CCSD(T) calculations, enabling DLPNO‐CCSD(T) calculations on large systems. ORCA is also well known for extensive support of theoretical spectroscopy methods and it includes relativistic approximations such as ZORA, DKH, and X2C as well as spin‐orbit coupling.

**FIGURE 4:**
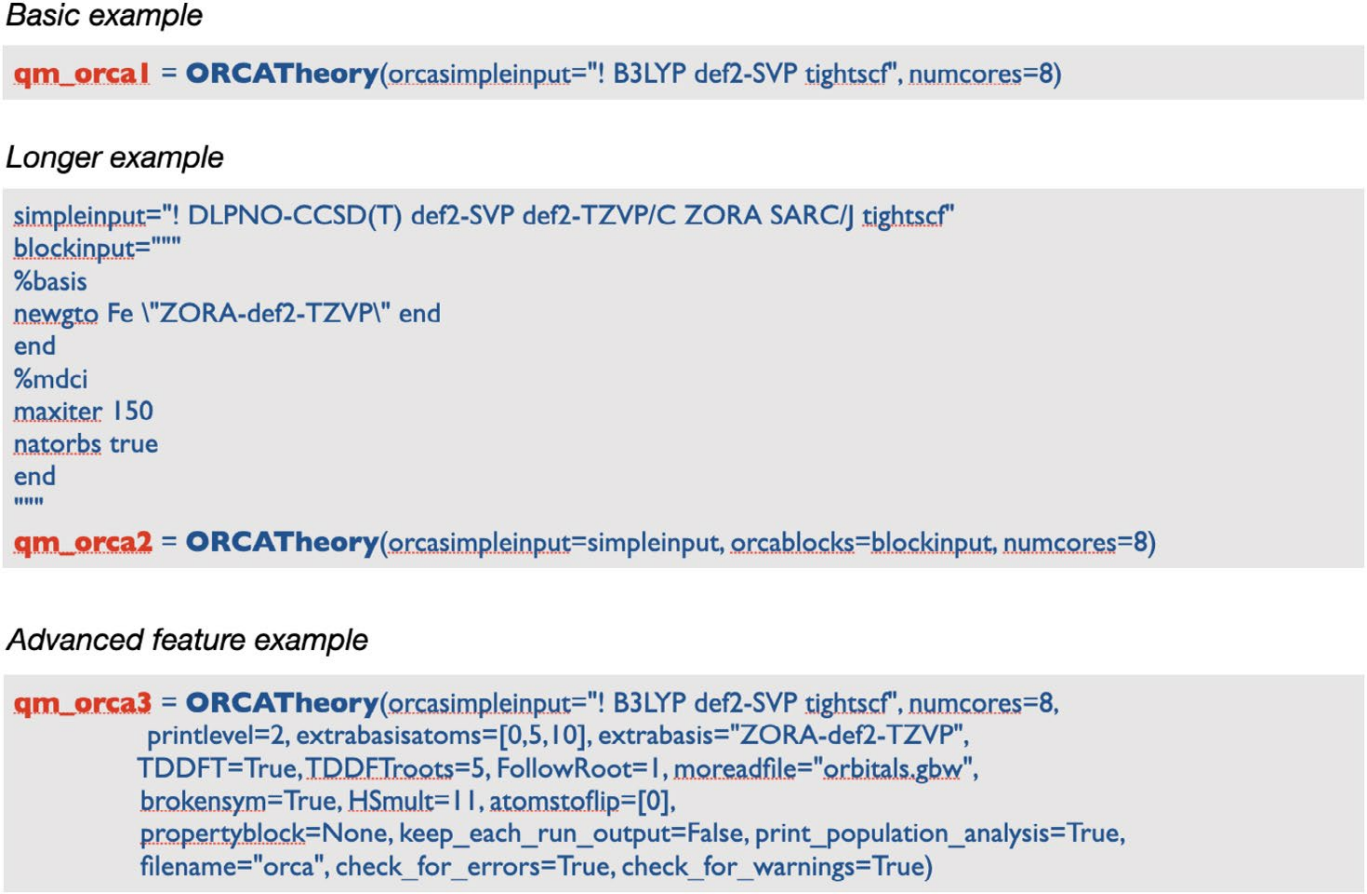
ASH input examples for defining **ORCATheory** objects of varying complexity. The first example defines a simple DFT theory specified as a single‐line string, the second shows how more control of the ORCA calculation can be achieved in a DLPNO‐CCSD(T) calculation setup, while the 3rd example shows how advanced keyword options in the interface can be used to activate a TDDFT excited‐state gradient calculation from a BS‐DFT ground state (while also activating various extra options).

ORCA has the most flexible interface in ASH, due in part to our long experience with the program, but also because it has a straightforward input‐syntax and a general availability of efficiently implemented electronic structure methods of both DFT and WFT type. The ORCA interface in ASH is highly flexible, and one can simply tell the interface what Hamiltonian, basis set, and other information should be present in the ASH‐created ORCA input file by providing text strings that define the simple‐input line/lines or block‐style input. Figure 4 shows three ways of creating **ORCATheory** objects, differing in complexity.

Additionally, the interface offers various options that allow convenient control over more complex basis set definition (e.g., specifying a specific basis set per element or per atom‐index), convenient broken‐symmetry solution control, TDDFT excited state gradient control, control over MO‐guess, and many other features, as shown in Figure 4. When running an ASH‐job using **ORCATheory**, ASH will first create an ORCA input file containing the current molecular coordinates as well as the specified input options, launch ORCA as a Python subprocess, wait until ORCA finishes execution, and then extract the energy and gradient (and sometimes Hessian or properties such as dipole moment, polarizability, and transition energies) from the outputfiles that ORCA generates. The interface to ORCA also supports point charges, allowing electrostatic embedding QM/MM.

**FIGURE 5:**
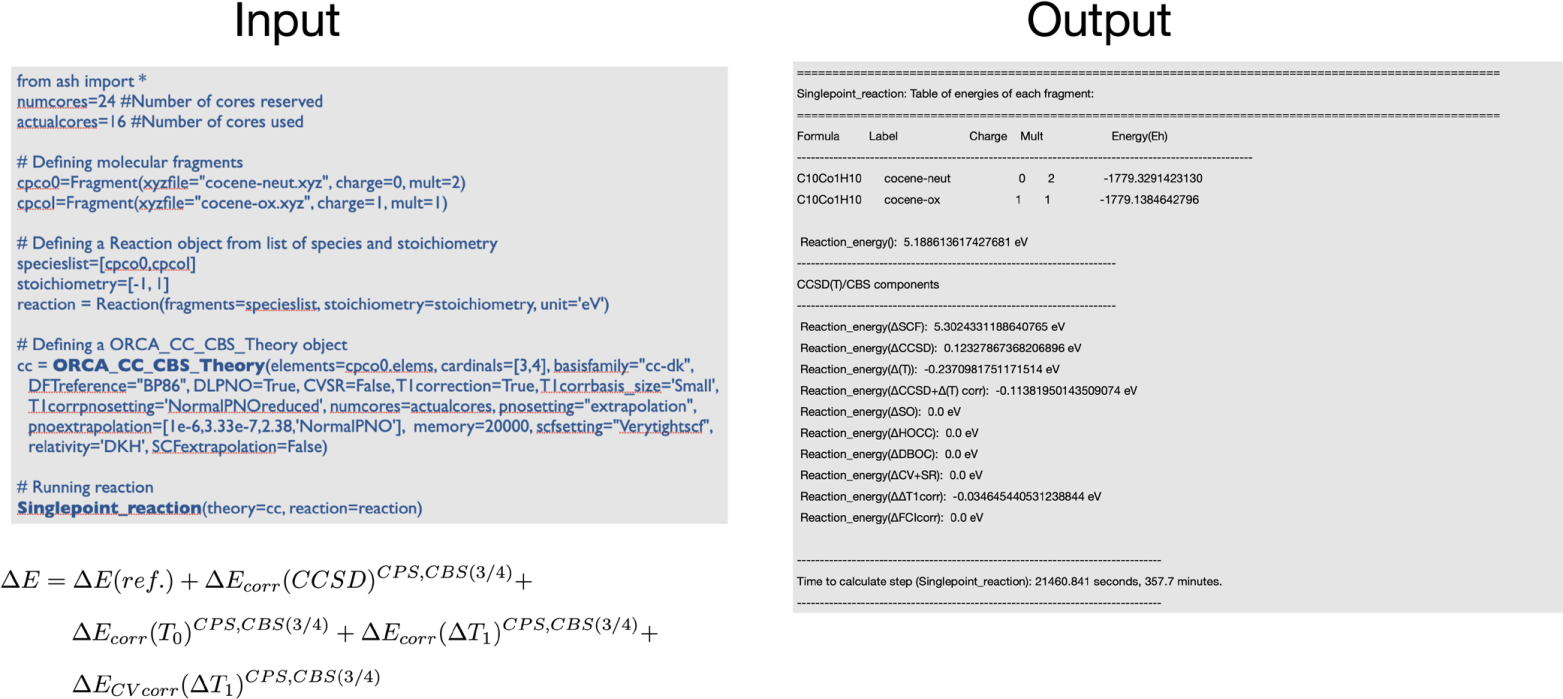
Example showing how the DLPNO‐CCSD(T)/CBS workflow as defined in Reference [37] can be defined for calculating the ionization energy of cobaltocene according to the multi‐theory energy expression shown.

In addition to **ORCATheory**, ASH also features a specialized theory class named **ORCA_CC_CBS_Theory**. This special interface implements a general CCSD(T)/CBS workflow, by instructing ORCA to carry out each step of a multi‐step composite‐method workflow that estimates the CCSD(T) or DLPNO‐CCSD(T) energy in the complete basis set limit, with or without additional corrections such as core‐valence, or post‐CCSD(T) corrections. For DLPNO methods, it is also possible to perform extrapolation to the complete PNO space (CPS) limit [36], and together this allows for convenient DLPNO‐CCSD(T)/CBS calculations that otherwise would require manually performing many individual energy calculations. This workflow was written into ASH as part of a DLPNO coupled cluster study on the ionization energy of cobaltocene and other metallocenes from our group [37]. Figure 5 shows the example input and output of using **ORCA_CC_CBS_Theory**.

Finally, the ORCA interface also features functions for conveniently extracting wavefunction information (MO coefficients, basis set, density matrix, 1 and 2‐electron integrals) from a JSON‐file created by the orca_2json helper program (part of ORCA). The ability of the interface to read in wavefunction information from ORCA even allows this information to be passed onto other programs. An ORCA‐calculated reference wavefunction can in this way, for example, be used to set up an advanced coupled cluster calculation using the ccpy coupled cluster program. We expect this type of interoperability between different programs at the wavefunction level to become more important in future ASH versions.

### 3.2 Interfaces to pySCF, gpu4pySCF, Block2 and Dice

pySCF [20, 21] is a general QM program undergoing rapid development due to its open‐source nature and an active developer community. It has an outer interface written in Python with all the computationally intensive parts (including the libcint integral library) written in C. The ASH interface to pySCF allows one to conveniently use the various powerful DFT and WFT‐based features in the program that can be combined with the geometry optimization, surface scans, NEB, numerical frequencies, QM/MM, MD, and features of ASH.

As pySCF is, in contrast to other programs, actually an extensive electronic structure method library with an essentially infinite number of options (all with different function calls) we have chosen to write the interface to pySCF in a rather different way than most other interfaces. It is almost impossible to extensively support every PySCF feature and ASH instead takes the approach of writing wrapper code around the most useful features that make them suitable for ASH workflows, QM/MM, and ONIOM coupling, and so forth. This makes it very easy to use PySCF within ASH for the most basic features but the drawback is that not every single feature inside the PySCF library can be supported.

**FIGURE 6:**
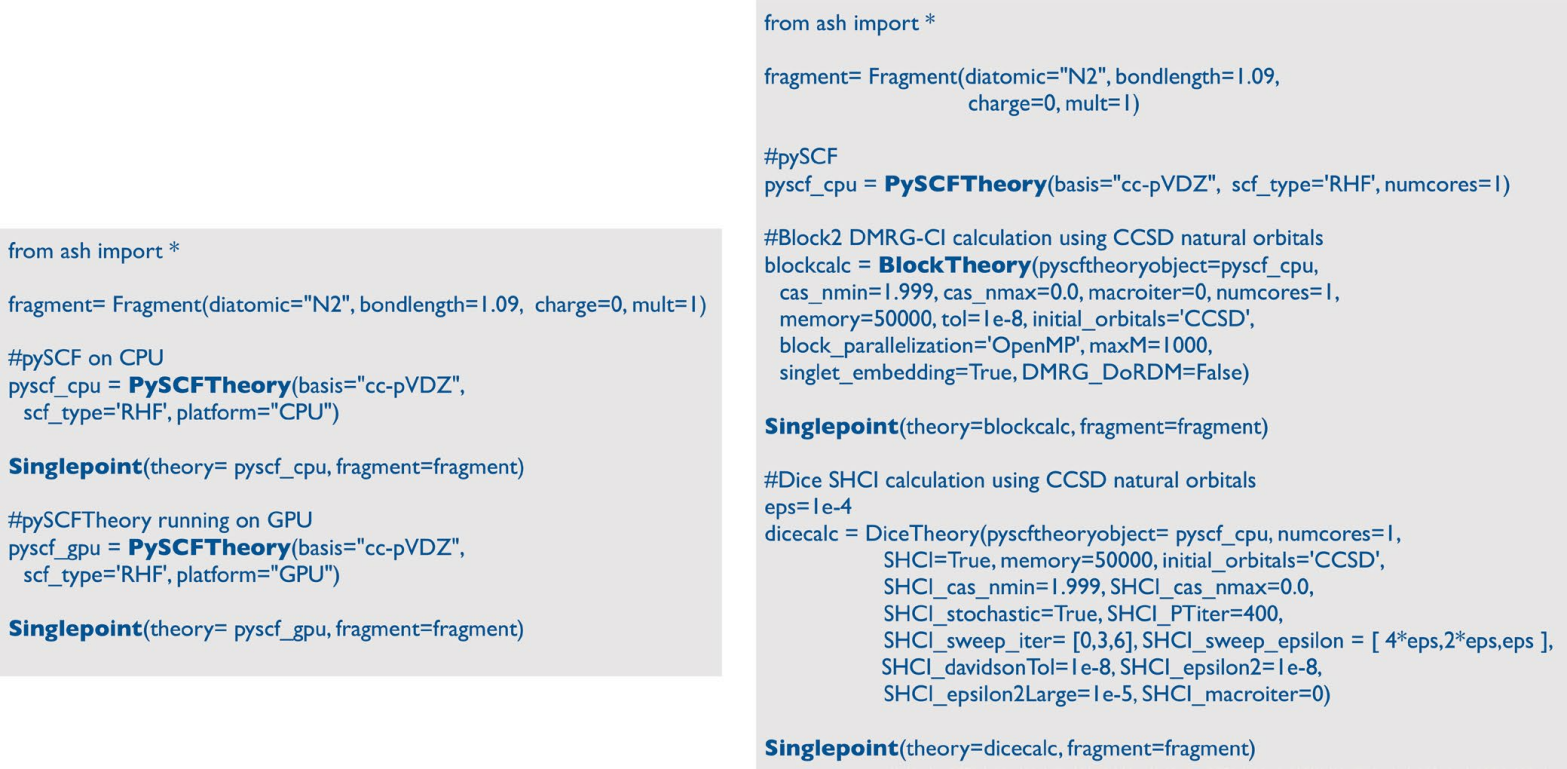
Left: Example showing the definition of a pySCFTheory object, running on either CPU or GPU. Right: Example of how a density matrix renormalization group (DMRG) or semi‐stochastic heat‐bath CI (SHCI) calculation can be easily started from a pySCFTheory object (using interfaces to the Block2 and Dice programs, respectively).

The **pySCFTheory** interface is also an interface to the gpu4pySCF plugin [30]. By simply changing the platform keyword from “cpu” to “gpu,” a HF or DFT calculation will be carried out by calling the CUDA‐specific gpu4pySCF code instead of the CPU‐specific pySCF code. Being able to run HF/DFT calculations on a GPU up to 30 times faster than the CPU code on a 32‐core CPU node offers considerable promise for performing faster HF/DFT calculations [31]. Figure 6 shows how a **PySCFTheory** object can be changed to run on either the CPU or the GPU.

The pySCF interface in ASH also serves the purpose of being used for interfaces to Block2 [38] (for DMRG calculations), Dice [39, 40] (for semi‐stochastic heat‐bath configuration interaction and quantum Monte Carlo). Both the DMRG and SHCI correlated wavefunction methods require an initial set of reference orbitals and integrals, which can be conveniently taken from the **PySCFTheory** object (making use of available pySCF plugins DMRGSCF and SHCI). The reference orbital choice is also easily modified in the ASH interface, and initial orbitals can, for example, be automatically changed to CCSD natural orbitals (calculated by pySCF) in either **BlockTheory** or **DiceTheory**, as shown in Figure 6.

### 3.3 MRCC, CFour, and CCpy

ASH features also interfaces to quantum chemistry programs specializing in coupled cluster theory. The CFour program features hand‐coded implementations of a variety of coupled cluster methods [25], including first and second derivatives, allowing for CC‐based structural optimizations and even analytical frequencies, as well as access to a variety of molecular properties at the CC level.

Meanwhile, the MRCC program [19] features an arbitrary‐order coupled cluster program (via code generation) that can compute the energy, gradient, and density matrices from pure CC methods of any order. MRCC also contains local natural orbital (LNO) methods [41] for cost reduction of CCSD(T) methods, enabling close to linear scaling of the CCSD(T) correlation energy as well as various DFT methods.

Both CFour and MRCC interfaces in ASH support energy and gradient computations, as well as point charge embedding, and can hence be used to run geometry optimizations, surface scans, NEB, and even MD simulations on its own or as part of a hybrid QM/MM scheme. We note that an advantage of using the CFour‐interface in ASH, instead of CFour directly, includes avoiding having to set up the manual Z‐matrix if the purpose is to perform geometry optimizations or relaxed surface scans at the CC level.

A very recent CC program, ccpy, written in Python, also has an interface in ASH, enabling easy use of various specialized types of coupled cluster methods based on the adaptive moment‐expansions [42, 43].

### 3.4 CP2K

The main focus of ASH has been on supporting interfaces to molecular quantum chemistry codes rather than periodic DFT codes, primarily utilizing planewaves to describe the solid‐state. With the interface to the CP2K program [26] we break this trend. CP2K contains a novel mixed Gaussian‐planewave method [44, 45] that enables fast DFT computations of the DFT energy and gradient with efficient parallelization, making molecular dynamics simulations at the DFT level possible. Because of the speed of CP2K it has become particularly popular for ab initio molecular dynamics studies. The program lacks, however, an internal‐coordinate geometry optimization algorithm or an eigenvector‐following algorithm for finding transition states, making the program perhaps less suitable for studying, for example, molecular systems in the gas phase or performing static reaction profile studies. The CP2K‐interface in ASH extends the capabilities of CP2K in this respect, enabling also alternative ways of performing QM/MM and ONIOM coupling.

### 3.5 xTB

The extended tight‐binding QM methods by Grimme and coworkers have reinforced the community's interest in fast, reasonably accurate semi‐empirical methods. The GFN1 [46] and GFN2 [24] Hamiltonians demonstrated that semi‐empirical methods could be capable of higher accuracy and scope than older methods. The most recent forthcoming g‐xTB method [47] aims even higher, offering close to DFT‐accuracy for energy surfaces.

ASH has included an interface to the xtb program since early on. The interface supports both disk‐based communication (writing coordinates to disk, calling xtb binary, and reading energy and gradients from files) and in‐memory communication (using the xtb‐python Python API). The former approach supports point charge embedding, thus enabling electrostatic embedding QM/MM in ASH. A separate preliminary interface to the g‐xTB method is also available. ASH also supports the modern tblite library for extended tight‐binding methods [22].

### 3.6 Other QM‐Program Interfaces

ASH features interfaces to programs with specific functionality such as GPU‐enabled DFT codes (Terachem [28], QUICK [48]), general semi‐empirical programs (MNDO, Sparrow [49]), 4‐component DFT molecular property calculations (ReSpect [50]), linear‐scaling wavelet‐based QM (BigDFT [51, 52]), auxiliary‐field QMC (ipie [53], Dice [39]). There are also interfaces to other popular QM‐codes: Dalton [54], Psi4 [55, 56], NWChem [27], Turbomole [57, 58], DFTB+ [59, 60], Gaussian. All interfaces support the calculation of the energy and usually an analytical gradient (when available), while only a few programs (or the respective interface) support the computation of an analytic Hessian. Many, but not all programs support point‐charge embedding; the ASH interface typically supports it if available. ASH also has interfaces to various smaller helper programs dedicated to computing, for example, DFT‐D4 [61, 62] (for D3/D4 dispersion corrections) or the gCP program (for geometric counterpoise corrections [63]).

### 3.7 OpenMM

A particularly important interface in ASH is the interface to OpenMM [14, 64]. Thus far, it is the only interface in ASH to a molecular mechanics (MM) program. OpenMM is an open‐source library, written in C++, CUDA, and Python, and is a popular program for running GPU‐based molecular dynamics simulations. It supports a variety of forcefields, integrators, and it can read legacy forcefield formats (e.g., CHARMM and Amber) as well as modern XML‐file formats. Being a library with both C++ and Python APIs, it is designed to be highly customizable. These features made OpenMM a particularly attractive candidate for interfacing to ASH, initially for its MM capabilities. The customizability of the library was also imperative in allowing QM/MM coupling according to the additive QM/MM approach, as well as running molecular dynamics with ASH, as described in the next sections.

## 4 Hybrid Theories

### 4.1 QM/MM, ONIOM

The hybrid QM/MM approach is arguably one the most important modeling principles in modern computational chemistry, which was recognized with the Nobel Prize given to Warshel, Levitt, and Karplus in 2013. It can be described as a very general way of focusing on the crux of a chemical problem, by dividing a large system into an important quantum mechanical (QM) region while the rest of the system (the environment) is described by a simpler theory, traditionally molecular mechanics (MM). QM/MM approaches come in various flavors and differ by the use of additive vs. subtractive energy expressions. A related approach is the ONIOM method, defined solely by a subtractive energy expression, which additionally allows the low‐level theory to be something other than an MM theory.

ASH features implementations of both the additive QM/MM approach as well as the subtractive ONIOM approach, using both mechanical and electrostatic embedding schemes. Both approaches can be thought of as hybrid approaches where 2 or more **Theory** objects, describing different parts of a molecular system, are combined to give a combined theory description of the system. ASH also features a hybrid approach where multiple **Theory** objects can be combined in arbitrary ways; this is referred to as **WrapTheory**. These different hybrid theories are described in the next sections.

### 4.2 QM/MM

The QM/MM energy expression implemented in ASH assumes an additive energy expression as popularized by Thiel and coworkers [65, 66, 67]:

$$E_{QM⁄MM}=E_{QM}+E_{MM}+E_{coupling}$$

$$E_{\text{coupling}}=E_{\text{elstat}}+E_{\mathrm{vdW}}+E_{\text{covalent}}$$

where *E*_*QM*_ is the QM‐energy of the QM‐region, *E*_*MM*_ is the MM‐energy of the MM‐region and *E*_*coupling*_ is the coupling between the QM and MM region. The coupling term accounts for electrostatic, short‐range vdW and covalent (bonded) interactions between the QM and MM regions. The *E*_*elstat*_ is calculated using mechanical embedding or electrostatic embedding traditionally. In practice, electrostatic embedding is usually the desired approach which means that *E*_*elstat*_ and *E*_*QM*_ are simultaneously calculated by the QM‐program. Additionally, the MM‐program calculates both *E*_*MM*_ and *E*_*vdw*_ and *E*_*covalent*_ terms simultaneously and so in practice the QM/MM energy is the sum of a modified *E*_*QM’*_ and *E*_*MM’*_ energy.

If the QM‐MM boundary crosses a covalent bond, special handling of the boundary is required. For the calculation of the QM energy, a linkatom is commonly introduced to cap the dangling bond of the QM region. In ASH, the linkatom is almost always hydrogen (although ASH also allows other elements, such as fluorine); the linkatom bond length is by default a fixed 1.09 Å (corresponding to a typical C—H bond), which works well for simple boundaries. This fixed distance can be changed or automatically determined by a scaled difference between QM1 and MM1 positions. For electrostatic embedding, special handling of the MM point charges is required when linkatoms are present. In ASH, we have implemented the popular charge‐shifting approach of Sherwood and coworkers [3] (default), where the charge on the MM1 atom (the MM atom bound to the QM1 atom at the boundary) is set to 0.0 and the original charge redistributed equally to MM atoms bound to it. In order to correct the dipole, a dipole correction is also typically applied (default in ASH). Also available is the redistributed charge and dipole (RCD) scheme by Truhlar and coworkers [68]. During the calculation of the QM system, the fictitious linkatoms experience forces; these forces should be projected onto the QM1 and MM1 atoms. Practical force projection schemes have utilized equations based on the chain or lever rule [3]. We have implemented both force projection schemes and found them to be similar. Also present at the QM‐MM boundary is the *E*_*covalen*t_ term, accounting for bonded interactions between QM and MM atoms. To avoid double‐counting interactions at the boundaries, the following MM bonded terms are deleted: the dihedral QM3‐QM2‐QM1‐MM, the angle QM2‐QM1‐MM1. As discussed by Sherwood [3] these deletions can be considered as an approximate linkatom energy correction.

**FIGURE 7:**
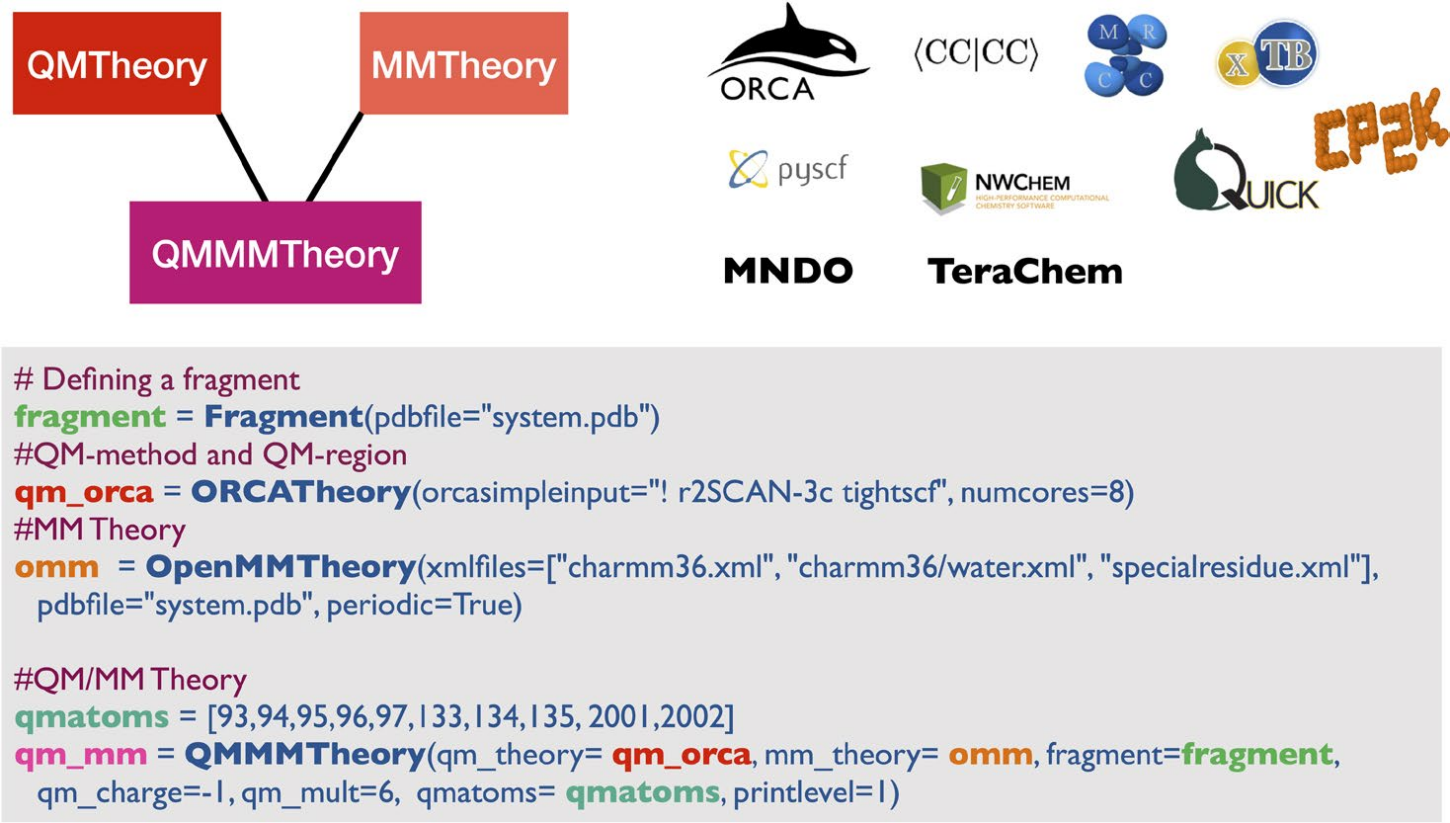
ASH input example showing the code for creating a **QMMMTheory** object that combines an **ORCATheory** and an **OpenMMTheory** object. The **ORCATheory** object is easily replaced by any other **Theory** object that supports pointcharge‐embedding for electrostatic embedding (current supported interfaces are shown), while mechanical embedding allows any **Theory** object. A defined **QMMMTheory** object can be used as input theory for any job‐type (as defined, e.g., in Figures 2 and 3).

As shown in Figure 7, setting up a QM/MM calculation in ASH (**QMMMTheory** class) is extremely simple as one only needs to combine a QM‐based **Theory** based object (here **ORCATheory**) and an MM‐based **Theory** object (**OpenMMTheory**) together in a **QMMMTheory** object, while specifying which atom indices are QM (with the rest being MM). Once a **QMMMTheory** object is defined it can be used as input to any of the main job‐functions in ASH. Upon dividing the system into regions, ASH will instruct OpenMM to delete the QM3‐QM2‐QM1‐MM and QM2‐QM1‐MM1 covalent terms in the forcefield at each QM‐MM boundary. Linkatoms are also automatically set up for covalent QM1‐MM1 boundaries and multiple linkatoms on the same QM atoms are allowed (though not necessarily recommended). By default, the program also exits if an unusual boundary is detected (i.e., if the bond does not correspond to a typical carbon–carbon bond boundary); however, unusual boundaries can be enabled if desired. The linkatom coordinates are then passed to the QM‐based **Theory** in each energy+gradient calculation. The total QM/MM gradient is defined for the whole system according to the energy expression above with the addition of the linkatom‐force projection being carried out. The total QM/MM gradient has the same dimensions as the coordinates of the whole system and can be used for any job‐type requiring a gradient: for example, geometry optimizations, nudged elastic band, and molecular dynamics.

### 4.3 ONIOM

Unlike the common additive QM/MM energy expression described above, the ONIOM method is defined by a subtractive expression [69]. The simplest method involves two levels of theory, low‐level (LL), and high‐level, then denoted 2‐layer ONIOM:

$$E_{\text{ONIOM}}=E_{12}^{\mathrm{LL}}+E_{1}^{\mathrm{HL}}-E_{1}^{\mathrm{LL}}$$

while 3 levels of theory, involving an extra medium‐level (ML) is the 3‐layer ONIOM method:

$$E_{\text{ONIOM}}=E_{123}^{\mathrm{LL}}+\left(E_{1}^{\mathrm{HL}}-E_{1}^{\mathrm{ML}}\right)+\left(E_{2}^{\mathrm{ML}}-E_{2}^{\mathrm{LL}}\right)$$

The purpose of the ONIOM layering, like in QM/MM, is to approximate a high‐level theory description of the whole system by a combination of a high‐level‐theory description of the important part of the system and a low‐level‐theory description of the environment part of the system. The main difference of a 2‐layer ONIOM method with respect to the additive QM/MM energy expression is that (i) a calculation of the whole system at the LL theory is required (E_12_^LL^ or E_123_^LL^) and (ii) the LL theory is not restricted to be a classical forcefield (MM) and (iii) the coupling between systems works a little differently.

It is helpful to think of the energy expression as a high‐level correction (E_1_^HL^−E_1_^LL^) to the whole‐system low‐level description (E_12_^LL^). A sensible division between subsystems must first be obtained, requiring linkatoms for covalent QM‐MM boundaries, as in QM/MM. If a sensible division is obtained, however, the LL theory in 2‐layer ONIOM is not limited to be an MM method, and could be a cheaper QM method from any interface (e.g., a DFT‐method or a semi‐empirical tight‐binding method like GFN2‐xTB) or even a machine‐learning interatomic potential (as shown later). During the calculation of the high‐level correction (E_1_^HL^−E_1_^LL^) one must compute the energy of Region 1 using both the low‐level and high‐level of theory. This correction can be performed on Region 1 alone or alternatively with point charges of Region 2 included in the form of electrostatic embedding (for E_1_^HL^ and E_1_^LL^ calculations). If no pointcharges are included and LL = MM, the method corresponds to a mechanical embedding scheme analogous to mechanical‐embedding additive QM/MM, while if pointcharges are included, the method is superficially similar to electrostatic embedding additive QM/MM. It is important to realize, however, that the actual coupling between regions occurs in the E_12_^LL^ calculation and the mechanical vs. electrostatic embedding schemes in ONIOM only serve the purpose of modifying how the high‐level correction term (E_1_^HL^−E_1_^LL^) is computed. Furthermore, if LL is a QM method, the coupling between regions is actually quantum mechanical, regardless of what embedding scheme is used to calculate the (E_1_^HL^−E_1_^LL^) term.

**FIGURE 8:**
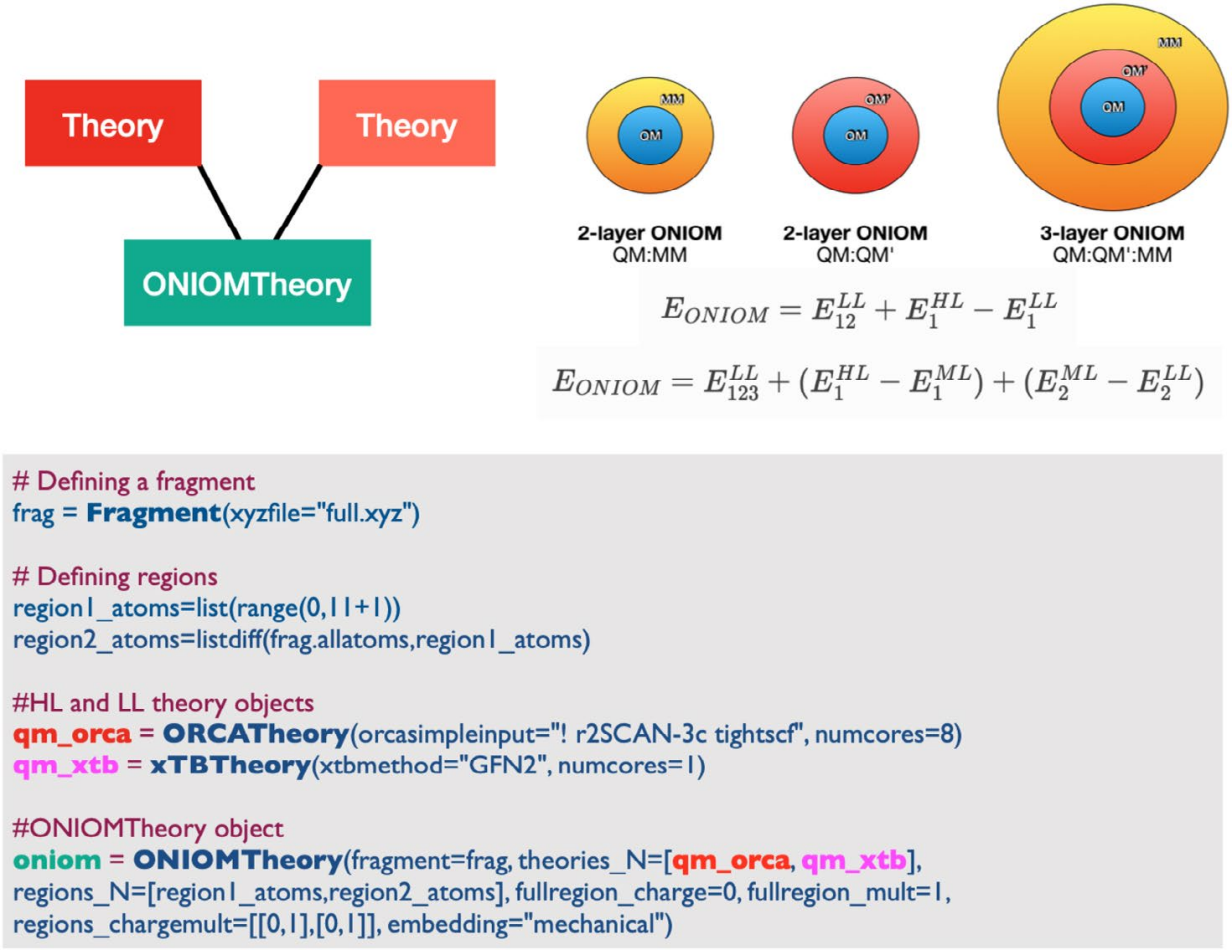
ASH input example showing the Python code for creating an **ONIOMTheory** object (here a 2‐layer QM:QM’ ONIOM), combining **ORCATheory** and **xTBTheory** objects. A defined **ONIOMTheory** object can be used as input‐theory for any job‐type (as defined, e.g., in Figures 2 and 3).

The primary advantage of the ONIOM method as implemented in ASH compared to most other ONIOM implementations is the general ability to combine different theory‐levels from different external program interfaces and apply those theory‐levels to different parts of a molecular system. Linkatoms, for capping QM subsystems, are automatically applied like in the QM/MM module, allowing for flexible boundary definitions. Both mechanical and electrostatic embedding schemes can be utilized, though atom charges may have to be pre‐defined if charges are not automatically available for the LL theory (if LL is not MM). Furthermore, both the LL and HL theories have to support point charge embedding in those cases. As shown in Figure 8, **ONIOMTheory** objects in ASH are very easy to set up and once defined, can be used to run geometry optimizations, molecular dynamics, surface scans, NEB, and so forth.

### 4.4 WrapTheory

**FIGURE 9:**
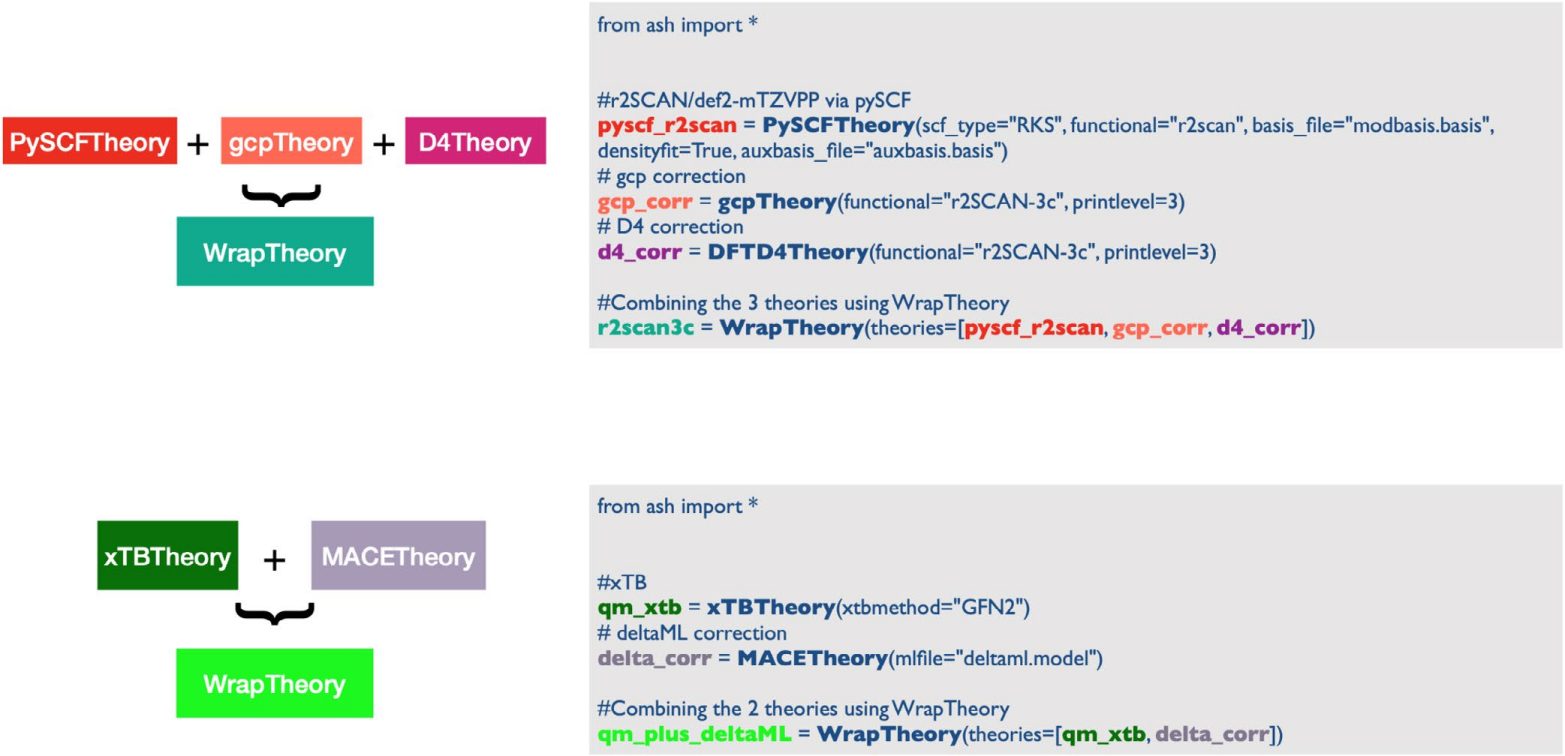
WrapTheory examples showing how one can (i) define the r^2^SCAN‐3c composite method manually using three different interfaces and (ii) define a Δ‐machine‐learning correction via an interface to a MACE machine‐learning interatomic potential to **xTBTheory**.

QM/MM and ONIOM methods apply multiple levels of theory to different regions of a molecular system. It is also possible to imagine applying multiple levels of theory to the same region. Anticipating the need for defining flexible theories that would be a sum (or a combination of sum and subtraction) of components from different sources, we implemented **WrapTheory**, a class that defines an energy expression as a sum of the energies (by default) from each theory defined. Such a hybrid theory object allows additional flexibility in performing multi‐scale modeling, on top of QM/MM and ONIOM, as they allow for defining additional energy terms as corrections. Figure 9 demonstrates the use of **WrapTheory** for 2 use‐cases. In one use‐case the r^2^SCAN‐3c composite method (not implemented in **PySCFTheory**), is defined as a sum of a **PySCFTheory** (defining r^2^SCAN with the correct basis set), **gcpTheory** (defining the geometric counterpoise correction) and **DFTD4Theory** (defining the dispersion correction). In another example, an **xTBTheory** level can be combined with a pretrained machine‐learning model that is defined to serve as an additive correction. The next section discusses machine‐learning theories in ASH further.

Importantly, any WrapTheory object in ASH (whatever its components) is just as valid a Theory‐object as any other and can thus be used for any of the main job‐functions (e.g., geometry optimizations, dynamics, NEB, etc.). This construct allows the user to easily define their own novel multi‐level theories, that are automatically compatible with most ASH job‐types.

## 5 Machine Learning Interatomic Potentials

Machine learning approaches are beginning to make a strong impact in computational chemistry. The field of machine‐learning interatomic potentials (MLIP) is a particularly promising area [70, 71], as MLIPs have been shown to be capable of sometimes replacing the expensive quantum chemical computation of the potential energy surface in favorable circumstances or at the very least going beyond the simplistic analytical classical MM potentials to more flexible reactive potentials. MLIPs can be thought of as force fields that avoid the use of fixed‐equation classical analytical formulas, replacing them with highly flexible neural networks or other architectures.

In addition to direct training and use of such potentials as an alternative to QM or MM, Δ‐learning approaches have also shown considerable promise. In Δ‐learning [72], the model can, for example, be trained to learn the difference between a low‐level (LL) and a high‐level (HL) QM treatment of a potential energy surface, resulting in a trained correction potential that can be used to correct the LL description. Since this approach would only try to learn the difference between 2 energy surfaces, instead of learning the energy surface from scratch, Δ‐learning requires much less training and can be thought of as a variant of physics‐informed ML. Use of the trained Δ potential, would, however, rely on the LL theory being used throughout a computation or simulation which would increase cost but perhaps favor robustness. ASH offers a lot of opportunities and flexibility for training MLIPs and Δ‐MLIPs, having interfaces to so many QM programs. These approaches show considerable promise for speeding up otherwise expensive QM calculations and simulations. With most functionality of ASH being theory‐agnostic, MLIP approaches fit neatly into the philosophy of the ASH program.

ASH currently offers interfaces to a few different neural network potential architectures, such as ANI models [73] (via TorchANI [74] and PyTorch libraries [75]), AIMNet2 [76] and AIMNet2‐NSE models [77] as well as the equivariant MACE [78] models. A recent interface to the Universal Model for Atoms (UMA) [79] foundational model potentials trained on the large OMol25 data set [80] is also available. Some of the interfaces enable the use of pre‐trained foundational models for most of the periodic table (e.g., UMA‐s‐1p1) while other interfaces also allow the training of bespoke models from the user's own data. An interface to the MLatom library [81] is also available that offers its own interfaces to various types of machine learning potentials as well as workflows to carry out the training.

While ASH is relatively new to the machine‐learning ecosystem, it already contains some functionality for aiding in training MLIPs, suitable at least for small data. It is straightforward to write ASH scripts for generating the molecular coordinate data: for example, from MD or Wigner ensembles or reaction path calculations from NEB (at many levels of theory) and subsequently calculate energies and gradients using the many QM‐program interfaces, and so forth. ASH even contains a function that can automatically prepare this training data for either MLIP or Δ‐MLIP training. A user‐trained, or alternatively a pre‐trained, foundational model, can then be as easily used as any other Theory level within ASH, as long as the model is capable of predicting a reliable energy and gradient for a molecular system with the correct elements. Finally, the ability to combine MLIPs within the ONIOM approximation or as a Δ correction to QM, QM/MM or ONIOM theories (via WrapTheory as shown in Figure 9) offers various unexplored opportunities, making ASH particularly powerful for combining traditional computational chemistry approaches and modern machine‐learning potentials within a multi‐scale modeling framework.

## 6 Geometry Optimizations, Surface Scans, and Reaction Paths

### 6.1 Geometry Optimizations

A geometry optimization is arguably the single most important job‐type in computational chemistry, allowing the exploration of minima on the Born‐Oppenheimer potential energy surface. While in its most basic form, a basic minimization algorithm such as steepest descent can easily be written to minimize geometries in Cartesian coordinates, in practice such an algorithm is too inefficient for molecular calculations. The combination of some suitable internal coordinate system with quasi‐Newton algorithms (such as L‐BFGS) is considered the state‐of‐the‐art approach. To avoid reinventing the wheel we have chosen to create interfaces to two powerful open‐source geometry optimization libraries: the geomeTRIC library [82] and the DL‐FIND library [83].

geomeTRIC is a Python library intended for molecular structure minimizations using various internal coordinates. It features a L‐BFGS minimization algorithm that can be combined with internal coordinate systems such as hybrid delocalized coordinates (HDLC) [84] as well as systems based on translation‐rotation coordinates (TRIC) [82]. The library supports constraints and frozen atoms and a restricted‐step partitioned rational function optimization (RS‐P‐RFO) algorithm [85] is available for transition‐state optimizations using a user‐supplied Hessian (or calculated numerically by the library). More recently implemented in ASH is an interface to the Fortran‐based DL‐FIND geometry optimization library. DL‐FIND became popular as an optimizer for QM/MM theories in the Chemshell program and features minimization algorithms and internal coordinate systems that work well for large systems (including delocalized, DLC, and hybrid delocalized, HDLC, internal coordinates). The DL‐FIND interface in ASH is more complicated (compared to geomeTRIC), relying on a C‐language layer as a bridge between Fortran and Python layers (using the libdlfind library https://github.com/digital‐chemistry‐laboratory/libdlfind, written by Kjell Jorner). The interface has been tested for optimizations (including QM/MM), transition‐state optimizations, nudged elastic band calculations, and dimer transition‐state searches. With DL‐FIND being entirely written in a compiled language, it may offer some computational advantages when optimizing systems with many degrees of freedom.

**FIGURE 10:**
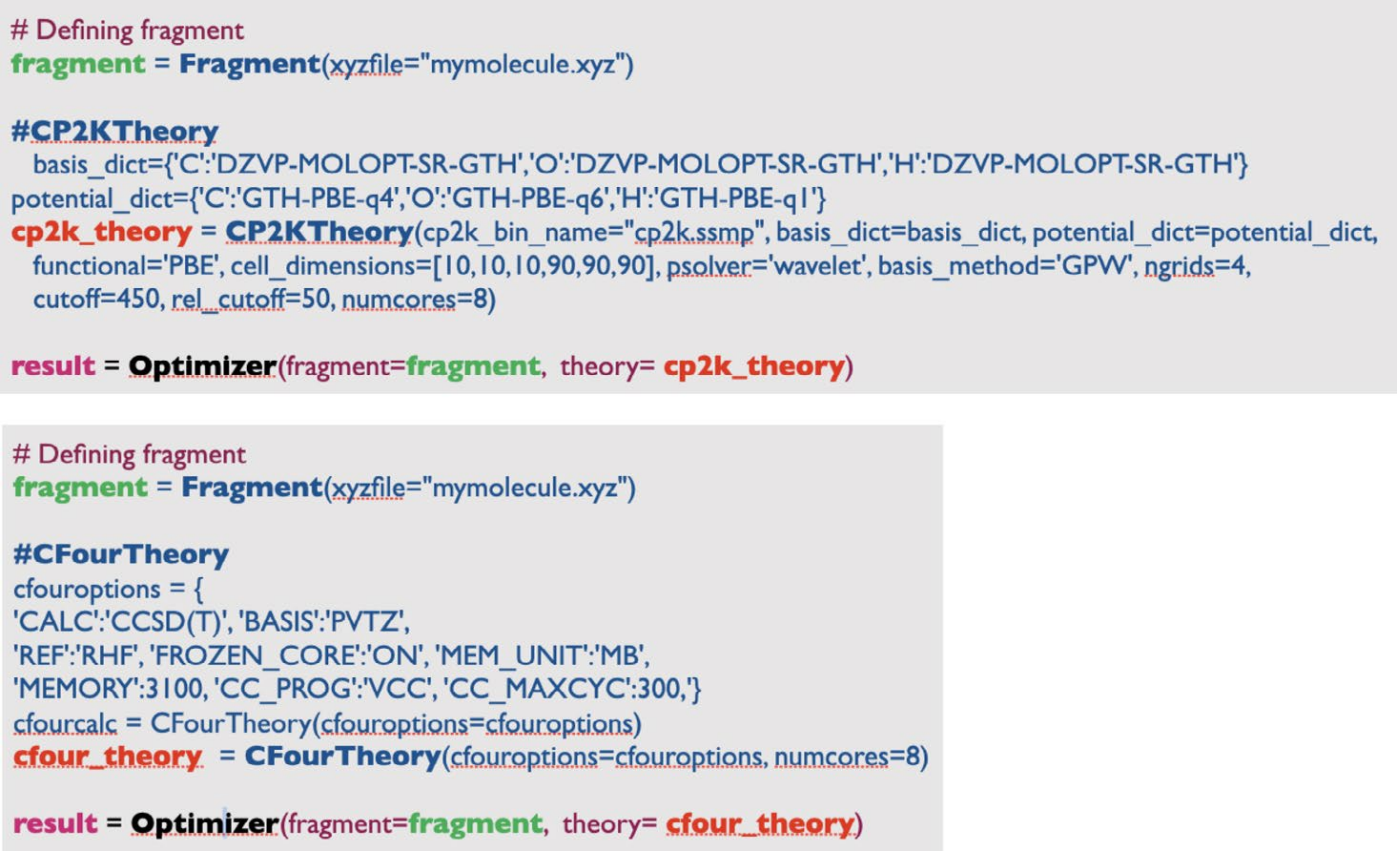
Example ASH scripts showing how internal coordinate geometry optimizations in ASH are easily performed utilizing electronic structure methods implemented in either CP2K (PBE with a mixed Gaussian‐planewave basis) or CFour (CCSD(T)/cc‐pVTZ). The optimization would be performed via the geomeTRIC library by default.

Geometry optimizations in ASH are performed by calling an Optimizer function, that is by default an alias to the ASH interface to the geomeTRIC library. Having an internal‐coordinate geometry optimizer outside the QM codes allows a common way to optimize molecular geometries using any theory level with an interface in ASH. As a specific example, using the interface to the CP2K program in ASH with the interface to the geomeTRIC optimization library allows the use of internal‐coordinate geometry optimization using CP2K, avoiding the limitation of Cartesian‐only coordinate optimization in CP2K, as shown in Figure 10. The figure also shows how geometry optimizations using coupled cluster methods are also easily performed using the interface to CFour, taking advantage of the availability of analytic gradients for coupled cluster methods in CFour, but using ASH avoids having to manually set up Z‐matrices (otherwise required in CFour).

As the optimizers in ASH are completely theory‐independent, any Theory object capable of producing an energy and gradient (for the whole system) can be used. This includes hybrid theories such as QM/MM, ONIOM, and WrapTheory. For large systems, one typically defines an active region by specifying a list of atom indices that should be active during a geometry optimization (the rest being frozen). ASH will then only provide the optimizer with the coordinates and gradient of the active region (while still requesting a calculation of the whole system).

In addition to the powerful internal‐coordinate based geomeTRIC and DL‐FIND optimizers, ASH also features simpler built‐in Cartesian‐coordinate based optimization algorithms, based on steepest descent, L‐BFGS or damped dynamics that have some limited use. A special L‐BFGS optimizer for **OpenMMTheory** objects for large systems is also available (from OpenMM library), only suitable for initial structural relaxations of large MM systems. Finally, ASH allows minimum energy crossing point optimizations, that is, finding the point where two energy surfaces (of different spin or symmetry) cross each other, based on the algorithm by Harvey et al. [86].

Analytical gradients of the QM‐methods are typically a prerequisite for efficient geometry optimizations. For many correlated wavefunction methods, analytical gradients are seldom available, due to the complicated equations or algorithms involved. Numerical gradients are the only choice in such cases, and ASH provides a NumGrad wrapper class that enables numerical‐gradient optimizations for any **Theory** object available in ASH.

### 6.2 Surface Scans, Reaction Paths and Transition‐State Searches

Relaxed and unrelaxed surface scans are also available in ASH. Relaxed surface scans utilize constraints on a specified geometric variable and then perform multiple constrained optimizations. Both one‐dimensional and two‐dimensional relaxed or unrelaxed scans can be performed using distance, angle, and dihedral coordinates, using geomeTRIC and DL‐FIND as optimizers.

ASH features a built‐in nudged elastic band (NEB) method [87, 88], available by an interface to the Knarr code by Vilhjálmur Ásgeirsson. The advantage of this NEB implementation is the recent rediscovery of the energy‐weighted springs NEB algorithm [89], that together with the climbing image variant [89], L‐BFGS [90], image‐dependent pair‐potential [91] interpolation and translation‐rotational invariance by quaternion algebra [92, 93] result in a particularly robust algorithm for molecular reactions. This was demonstrated in a recent NEB study as part of the C++ implementation work in the ORCA code [94]. This original Python‐based implementation of the method in Knarr when interfaced in ASH is arguably even more useful, as it makes the NEB method available to any QM‐method (or MLIP) with an ASH‐interface, and allows, for example, hybrid QM/MM NEB path calculations to be performed. Also introduced in the same NEB study [94], is the NEB‐TS method, that deliberately reduces expensive NEB iterations to a smaller number via higher convergence thresholds and then selects the crudely converged climbing image geometry and approximate Hessian for a P‐RFO transition‐state optimization [95]. In ASH, a similar NEB‐TS method has been implemented, combining the Knarr‐based NEB code, with the RS‐PRFO method [85] in geomeTRIC with various possible Hessian approximations available (including partial Hessians).

## 7 Numerical Frequency and Vibrational Spectroscopy

The calculation of the nuclear Hessian is critical for characterization of energy surface stationary points as well as the calculation of vibrational properties. It can be formulated as a numerical problem of energies and analytic gradients at displaced geometries or alternatively as a fully analytical second‐derivative problem. While an analytical Hessian implementation is often preferable, it is rarely available for complex electronic structure methods and is often memory‐demanding. Furthermore, a general QM/MM or ONIOM strategy to the Hessian would typically require a numerical approach. While numerical Hessians are often available in QM programs, they sometimes differ in their implementations.

In ASH, we have implemented a general numerical Hessian approach that can be used for any Hamiltonian available. It supports both 1‐point and 2‐point formulas, partial Hessian evaluation (based on selected atom indices), and parallelization over displacement calculations. The user simply calls the NumFreq function, provides an input **Fragment** object and an input **Theory** object and possibly some optional keyword arguments (e.g., 1‐point or 2‐point option, input masses, number of CPU cores, parallelization strategy, partial Hessian definition, etc.). In the default serial mode, NumFreq will request the calculation of each displacement sequentially; if CPU‐core information has been provided to the requested Theory object, the external QM code will run each displacement in parallel‐mode (using its own parallelization strategy). Alternatively, NumFreq supports a more efficient parallelization mode, where multiple displacements can be run simultaneously (in random order) using the available CPU‐cores, using the Python multiprocessing library. Typically, external‐code parallelization would be turned off when this strategy is used.

Following the completion of the Hessian calculation, the Hessian is written to disk (for user convenience) and is diagonalized. Projection of translational and rotational modes is carried out using functionality from the geomeTRIC library. Next, a rigid‐rotor harmonic oscillator analysis is carried out and thermochemical quantities printed using the vibrational frequencies, geometry, and mass information. ASH supports a quasi‐harmonic approach, such as the strategy by Grimme and Truhlar for correcting the vibrational entropy from low‐frequency modes.

ASH also supports the calculation of IR and Raman intensities/activities. Infrared intensities require the calculation of dipole derivatives, which can be derived from the dipole moment for each displacement, which is typically automatically available from the energy‐calculation output of the external code. Currently, the following program interfaces in ASH support IR intensities: ORCA, xTB, CFour, MRCC, pySCF, Dice, and Block2. Raman activities require polarizability derivatives which, in turn, require the calculation of polarizability for each displacement. The polarizability is a more expensive property, typically not calculated by default (needs to be requested), and not always available in any QM program. Currently, the ORCA, CFour, and pySCF interfaces in ASH support Raman activities.

Vibrational or ensemble averaging of energies and properties is often required, for example, for accurate spectral simulations. The calculation of vibrational effects from higher‐order energy derivatives are beyond the scope of this article (and ASH for the moment) while ensemble averaging from an MD simulation are typically too expensive. A popular alternative in the excited state dynamics community is the calculation of the Wigner ensemble which is an ensemble of geometries derived from the harmonic nuclear Hessian. Using a function to generate the Wigner ensemble from the geomeTRIC library, ASH can easily provide a Wigner ensemble of user‐specified N geometries at a chosen temperature for any method capable of providing an analytical or numerical Hessian (or an external Hessian read from file). The ensemble of geometries can then be used within ASH for vibrational averaging of various properties (by performing additional calculations on each geometry).

## 8 Dynamics and Free Energy Simulations

ASH allows Born‐Oppenheimer (BO) molecular dynamics simulations to be performed in a general way. This is achieved via the interface to the OpenMM library that includes various integration algorithms, thermostats, and barostats. These algorithms simply propagate atom dynamics based on the atomic forces, and since ASH can compute atomic forces in many different ways, ASH can support BO‐based molecular dynamics for essentially any level of theory.

When an OpenMMTheory object (i.e., an MM theory) is used as input, ASH simply calls the OpenMM library to carry out the MM MD simulation without any overhead. For non‐MM Hamiltonians, ASH will, however, drive the simulation timestep‐by‐timestep and in each timestep the atomic forces in an OpenMM simulation object are updated via a separate energy+gradient computation using the requested theory. This general strategy allows any interface to a QM or MM program (or even machine‐learning potential) to be used to propagate a dynamics trajectory, as long as the interface supports the calculation of an energy and gradient. While this approach, by necessity, introduces some overhead (due to data‐exchange), this is typically only on the order of 100–300 ms, which we consider a small price to pay for the generality of running MD simulations at any level of theory.

Enhanced sampling and free energy simulations are an especially important area. The OpenMM library supports various forms of restraints (including user‐created ones), and umbrella sampling simulations can easily be performed. ASH also supports the native well‐tempered metadynamics (WTM) implementation in OpenMM, allowing for easy‐to‐setup metadynamics using any level of theory in ASH. Additionally, thanks to an available OpenMM‐Plumed interface, most of the enhanced sampling methods and collective variables within the Plumed library (including metadynamics and OPES) are directly available and can be used within ASH. A third‐party interface between ASH and the adaptive‐sampling library from Ochsenfeld and coworkers [96] has also recently become available, enabling ASH to use various advanced enhanced sampling algorithms such as the (extended‐system) adaptive biasing force methods (ABF, eABF), their combinations with WTM and OPES, free‐energy NEB, and others [96, 97, 98, 99, 100, 101, 102].

Most importantly, all ASH Theory levels, including hybrid **QMMMTheory**, **ONIOMTheory**, and **WrapTheory** levels, can be used to run molecular dynamics or free energy simulations. This gives a very high degree of flexibility, allowing simulations at multiple scales and with multiple theories. With modern MLIP potentials becoming more and more accurate, their combination within a hybrid framework (e.g., QM/MM, ONIOM, and Δ‐learning) and use with advanced sampling algorithms, will allow lots of opportunities for future novel simulation strategies.

Finally, conformational sampling based on the CREST metadynamics‐based algorithm [103] by Grimme and coworkers has become popular. The algorithm is typically used via the extended tight‐binding methods from the Grimme group. ASH features a general interface to the CREST program [104] that allows any level of theory in ASH to be used for the metadynamics‐based conformational sampling or other algorithms implemented inside CREST.

## 9 Other Features

### 9.1 Workflows for Setting up Biomolecular and Explicit Solvation Systems

In addition to featuring flexible interfaces to many QM, MM, and ML methods, hybrid coupling methods such as QM/MM and ONIOM, and the ability to perform minimization and dynamics, ASH also features various ways of setting up complex systems as well as many pre‐programmed workflows.

A common user‐based bottleneck of performing a QM/MM study of an enzymatic reaction, for example, is how to prepare the system prior to running the QM/MM minimizations or dynamics simulations. A biomolecular system ready for running advanced multi‐scale simulations requires first an appropriately set up system, with all atoms present (often missing from experimental x‐ray structures), with an appropriate solvation shell surrounding the biomolecule (as well as filling empty cavities), and neutralization of any non‐zero charge present (ideally accounting for ion concentrations as well). This setup also requires the choice of an appropriate biomolecular force field as well as accounting for missing force field parameters associated with inorganic or organic molecular species present in the system. Finally, an appropriate equilibration at the MM level is typically also considered important before starting any more complex multi‐scale modeling simulations. Thanks to features available as part of the OpenMM ecosystem, biomolecular system setup is a lot easier, via the Modeller and pdbfixer functionality. In ASH, we have furthermore wrapped many of these features together in easy‐to‐use functions enabling almost automatic initial hydrogenation, solvation, minimization, initial warm‐up MD, as well as NPT equilibration. The functions are directly callable sequentially, even within a single ASH script, and can considerably expedite the system setup prior to starting, for example, QM/MM simulations. All in all, this results in a common working environment for both initial system setup and production multi‐scale simulations, as opposed to switching between multiple programs for these steps.

The modeling of solvated molecules, similarly, requires a setup process that can also involve considerable complexity, especially if the solvent is not water. Via an interface to the Packmol [105] program, the process to start explicit solvation dynamics studies (regardless of chosen theory) becomes a lot easier.

The ASH documentation (hosted at https://ash.readthedocs.io) contains several tutorials dedicated to showing by example how to carry out various complex tasks in ASH such as: setting up QM/MM models for a metalloprotein, setting up explicit solvent MM and QM/MM MD simulations, performing 1D and 2D metadynamics simulations (QM or QM/MM), how to select appropriate QM/MM boundaries in proteins, how to set up your own workflows, high‐level WFT workflows, analysis of densities from correlated wavefunction from various QM programs, and so forth.

### 9.2 Post‐Processing of Calculations

In addition to carrying out calculations and simulations, ASH also offers ways of expediting various types of post‐processing of computational chemistry data. Density analysis of WFT or DFT calculations can conveniently be performed using the information obtained from various QM programs. ASH offers internal tools to convert wavefunction files to Molden‐files [106] which can be automatically fed to the Multiwfn program [107] to produce Cube‐files of various types of densities. In the future we also hope to support efforts to standardize exchange of wavefunction data, such as the TREXIO format [108].

ASH offers a convenient wrapper around the Matplotlib library allowing easy plotting of various ASH‐job results such as reaction profiles and surface scans. The output of spectroscopy calculations (e.g., IR, UV–VIS, XAS) can be line‐broadened (Gaussian, Lorentzian, Voigt) before plotting.

## 10 Conclusions

ASH was conceived a few years ago and was open‐sourced and made freely available in 2022 on Github (https://github.com/RagnarB83/ash). It has been utilized in a diverse set of studies from our research group. Most notably, all our latest QM/MM studies on the metalloenzymes nitrogenase [109, 110, 111] as well as FeFe and NiFe hydrogenases [112, 113], utilize ASH using interfaces to ORCA and OpenMM. The interface to OpenMM was additionally utilized for protein setup and long‐time‐scale GPU‐based classical MM simulations to understand the binding of an inorganic Mo/Cu cluster to the Orange protein [114]. ASH is, however, far from being limited to biomolecular QM/MM studies. Nudged elastic band calculations using interfaces to xTB and ORCA were used to shed light on borrowing hydrogen reactions of organometallic catalysts [115]; and ASH has also been used to expedite more complicated calculations based on correlated wavefunction theory. In our systematic DLPNO coupled cluster study on the ionization energies of cobaltocene and other metallocenes, we utilized the ORCA interface for running DLPNO‐CCSD(T) calculations and additionally implemented an automatic multistep workflow for convenient DLPNO‐CCSD(T) extrapolation workflows to achieve the CBS and PNO limit [37]. In our joint study of the diradical character of dialumenes with the Kraemer group, the interfaces to various correlated wavefunction programs: Block, Dice, CFour, MRCC, and ORCA conveniently enabled a systematic study of different correlated wavefunction methods and DFT methods for both energy and density comparisons [116].

Other researchers have found ASH useful in their science. van Stappen and coworkers have used ASH for setting up new protein models and performing QM/MM spectroscopic studies of Cu proteins such as azurin variants [117, 118, 119]. Sun and coworkers used ASH to perform QM/MM calculations of phenylalanine ammonia lyase variants [120] while Arnold and coworkers used the program to explore a nonnative alkylation mechanism of β‐subunit of tryptophan synthase [121]. Zhu and coworkers used the NEB module in ASH to explore accelerating NEB calculations with an equivariant neural network potential [122] while Greene‐Diniz et al. used the NEB module with the pySCF interface of ASH to explore the minimum energy path of a complex reaction [123].

ASH is in an active state of development and many future developments can be foreseen. Polarizable QM/MM is an important development in multi‐scale modeling, allowing one to go beyond the one‐sided polarization of standard electrostatic embedding QM/MM. Unfortunately, due to the nature of the back‐and‐forth polarization, many polarizable embedding strategies require modification of the QM codes themselves, which would mean polarizable QM/MM would be restricted to particular QM codes rather than being generally available. The availability of CPPE [124], a polarizable embedding library from Scheurer, Kongsted, and coworkers, offers a promising path forward, enabling easier QM code modifications. Additionally, the simpler Drude‐polarizable strategy offers a simpler general polarizable QM/MM scheme (requiring only point charge support for the QM code) that we hope to implement into ASH in the future.

Machine‐learning functionality in ASH is also being actively explored. The program already features useful interfaces to, for example, MACE, TorchANI, AimNet2, Fairchem (UMA), and Mlatom, and some tools to help automate the training of MLIP potentials. ASH is already a promising platform for training and using MLIPs and Δ‐MLIPs due to available interfaces to many QM‐programs and the theory‐agnostic nature of ASH job‐types. ASH could also be used to develop active learning workflows. We are currently exploring implementation of machine‐learning tools to expedite various type of computational chemistry jobs and multi‐scale simulations.

Finally, support for periodic boundary conditions (PBC) in ASH is in an exploratory state of development. The OpenMM interface in ASH directly supports PBCs at the MM level (via the OpenMM library), and selected QM‐programs such as CP2K natively support PBCs. ASH currently does not feature a general QM/MM PBC strategy, meaning most QM codes will see only a finite set of point charges of the simulation box or sphere, but a more general QM/MM PBC implementation is in progress. Better support for computations of solid‐state systems, including new interfaces is also planned.

## Conflicts of Interest

The author declares no conflicts of interest.

## Data Availability

Data sharing not applicable to this article as no datasets were generated or analysed during the current study.

## References

[1] S. Lehtola , “A Call to Arms: Making the Case for More Reusable Libraries,” Journal of Chemical Physics 159, no. 18 (2023): 180901, 10.1063/5.0175165. 37947507

[2] A. H. Larsen , J. J. Mortensen , J. Blomqvist , et al., “The Atomic Simulation Environment—A Python Library for Working With Atoms,” Journal of Physics. Condensed Matter 29, no. 27 (2017): 273002, 10.1088/1361-648X/aa680e. 28323250

[3] P. Sherwood , A. H. de Vries , M. F. Guest , et al., “QUASI: A General Purpose Implementation of the QM/MM Approach and Its Application to Problems in Catalysis,” Journal of Molecular Structure: THEOCHEM 632, no. 1 (2003): 1–28, 10.1016/S0166-1280(03)00285-9.

[4] S. Metz , J. Kästner , A. A. Sokol , T. W. Keal , and P. Sherwood , “ChemShell—A Modular Software Package for QM/MM Simulations,” Wiley Interdisciplinary Reviews: Computational Molecular Science 4, no. 2 (2014): 101–110, 10.1002/wcms.1163.

[5] Y. Lu , M. R. Farrow , P. Fayon , et al., “Open‐Source, Python‐Based Redevelopment of the ChemShell Multiscale QM/MM Environment,” Journal of Chemical Theory and Computation 15, no. 2 (2019): 1317–1328, 10.1021/acs.jctc.8b01036. 30511845

[6] J. Steinmetzer , S. Kupfer , and S. Gräfe , “Pysisyphus: Exploring Potential Energy Surfaces in Ground and Excited States,” International Journal of Quantum Chemistry 121, no. 3 (2021): e26390, 10.1002/qua.26390.

[7] M. J. Field , “The pDynamo Program for Molecular Simulations Using Hybrid Quantum Chemical and Molecular Mechanical Potentials,” Journal of Chemical Theory and Computation 4, no. 7 (2008): 1151–1161, 10.1021/ct800092p. 26636368

[8] M. J. Field , “pDynamo3 Molecular Modeling and Simulation Program,” Journal of Chemical Information and Modeling 62, no. 23 (2022): 5849–5854, 10.1021/acs.jcim.2c01239. 36449463

[9] J. Řezáč , “Cuby: An Integrative Framework for Computational Chemistry,” Journal of Computational Chemistry 37, no. 13 (2016): 1230–1237, 10.1002/jcc.24312. 26841135

[10] S. Martí , “QMCube (QM3): An All‐Purpose Suite for Multiscale QM/MM Calculations,” Journal of Computational Chemistry 42, no. 6 (2021): 447–457, 10.1002/jcc.26465. 33337551

[11] D. G. A. Smith , D. Altarawy , L. A. Burns , et al., “The MolSSI QCArchive Project: An Open‐Source Platform to Compute, Organize, and Share Quantum Chemistry Data,” Wiley Interdisciplinary Reviews: Computational Molecular Science 11, no. 2 (2021): e1491, 10.1002/wcms.1491.

[12] E. Berquist , A. Dumi , S. Upadhyay , et al., “Cclib 2.0: An Updated Architecture for Interoperable Computational Chemistry,” Journal of Chemical Physics 161, no. 4 (2024): 042501, 10.1063/5.0216778. 39051837

[13] J. P. Pederson and J. G. McDaniel , “PyDFT‐QMMM: A Modular, Extensible Software Framework for DFT‐Based QM/MM Molecular Dynamics,” Journal of Chemical Physics 161, no. 3 (2024): 034103, 10.1063/5.0219851. 39007371

[14] P. Eastman , J. Swails , J. D. Chodera , et al., “OpenMM 7: Rapid Development of High Performance Algorithms for Molecular Dynamics,” PLoS Computational Biology 13, no. 7 (2017): e1005659, 10.1371/journal.pcbi.1005659. 28746339 PMC5549999

[15] C. R. Harris , K. J. Millman , S. J. van der Walt , et al., “Array Programming With NumPy,” Nature 585, no. 7825 (2020): 357–362, 10.1038/s41586-020-2649-2. 32939066 PMC7759461

[16] A. B. Yoo , M. A. Jette , and M. Grondona , “SLURM: Simple Linux Utility for Resource Management,” in Job Scheduling Strategies for Parallel Processing. Series Eds.; Lecture Notes in Computer Science, vol. 2862, ed. D. Feitelson , L. Rudolph , U. Schwiegelshohn , G. Goos , J. Hartmanis , and J. van Leeuwen (Springer Berlin Heidelberg, 2003), 44–60, 10.1007/10968987_3.

[17] “The ORCA *program system ‐ Neese ‐* 2012 *‐ WIREs Computational Molecular Science ‐ Wiley Online Library* ,” accessed April 12, 2026, 10.1002/wcms.81?casa_token=vz-TK4d1K1MAAAAA:hucMVkYIWw7p_O7I3MkAbVxkTWJxWvSjlivhgewf3qyYdXdyVfQO4QPzws6zDAgiiy_0bBBnCDrmsVg.

[18] F. Neese , “Software Update: The ORCA Program System—Version 6.0,” Wiley Interdisciplinary Reviews: Computational Molecular Science 15, no. 2 (2025): e70019, 10.1002/wcms.70019.

[19] M. Kállay , P. R. Nagy , D. Mester , et al., “The MRCC Program System: Accurate Quantum Chemistry From Water to Proteins,” Journal of Chemical Physics 152, no. 7 (2020): 074107, 10.1063/1.5142048. 32087669

[20] Q. Sun , X. Zhang , S. Banerjee , et al., “Recent Developments in the PySCF Program Package,” Journal of Chemical Physics 153, no. 2 (2020): 024109, 10.1063/5.0006074. 32668948

[21] Q. Sun , T. C. Berkelbach , N. S. Blunt , et al., “PySCF: The Python‐Based Simulations of Chemistry Framework,” Wiley Interdisciplinary Reviews: Computational Molecular Science 8, no. 1 (2018): e1340, 10.1002/wcms.1340.

[22] A. Katbashev , M. Stahn , T. Rose , et al., “Overview on Building Blocks and Applications of Efficient and Robust Extended Tight Binding,” Journal of Physical Chemistry. A 129, no. 10 (2025): 2667–2682, 10.1021/acs.jpca.4c08263. 40013428

[23] C. Bannwarth , E. Caldeweyher , S. Ehlert , et al., “Extended Tight‐Binding Quantum Chemistry Methods,” Wiley Interdisciplinary Reviews: Computational Molecular Science 11, no. 2 (2021): e1493, 10.1002/wcms.1493.

[24] C. Bannwarth , S. Ehlert , and S. Grimme , “GFN2‐xTB—An Accurate and Broadly Parametrized Self‐Consistent Tight‐Binding Quantum Chemical Method With Multipole Electrostatics and Density‐Dependent Dispersion Contributions,” Journal of Chemical Theory and Computation 15, no. 3 (2019): 1652–1671, 10.1021/acs.jctc.8b01176. 30741547

[25] D. A. Matthews , L. Cheng , M. E. Harding , et al., “Coupled‐Cluster Techniques for Computational Chemistry: The CFOUR Program Package,” Journal of Chemical Physics 152, no. 21 (2020): 214108, 10.1063/5.0004837. 32505146

[26] T. D. Kühne , M. Iannuzzi , M. Del Ben , et al., “CP2K: An Electronic Structure and Molecular Dynamics Software Package ‐ Quickstep: Efficient and Accurate Electronic Structure Calculations,” Journal of Chemical Physics 152, no. 19 (2020): 194103, 10.1063/5.0007045. 33687235

[27] E. Aprà , E. J. Bylaska , W. A. de Jong , et al., “NWChem: Past, Present, and Future,” Journal of Chemical Physics 152, no. 18 (2020): 184102, 10.1063/5.0004997. 32414274

[28] S. Seritan , C. Bannwarth , B. S. Fales , et al., “TeraChem: A Graphical Processing Unit‐Accelerated Electronic Structure Package for Large‐Scale Ab Initio Molecular Dynamics,” Wiley Interdisciplinary Reviews: Computational Molecular Science 11, no. 2 (2021): e1494, 10.1002/wcms.1494.

[29] Y. Miao and K. M. Merz, Jr. , “Acceleration of Electron Repulsion Integral Evaluation on Graphics Processing Units via Use of Recurrence Relations,” Journal of Chemical Theory and Computation 9, no. 2 (2013): 965–976, 10.1021/ct300754n. 26588740

[30] R. Li , Q. Sun , X. Zhang , and G. K.‐L. Chan , “Introducing GPU Acceleration Into the Python‐Based Simulations of Chemistry Framework,” Journal of Physical Chemistry. A 129, no. 5 (2025): 1459–1468, 10.1021/acs.jpca.4c05876. 39846468 PMC11808769

[31] X. Wu , Q. Sun , Z. Pu , et al., “Enhancing GPU‐Acceleration in the Python‐Based Simulations of Chemistry Framework,” arXiv July 22 (2024), 10.48550/arXiv.2404.09452.

[32] C. Riplinger and F. Neese , “An Efficient and Near Linear Scaling Pair Natural Orbital Based Local Coupled Cluster Method,” Journal of Chemical Physics 138, no. 3 (2013): 034106, 10.1063/1.4773581. 23343267

[33] C. Riplinger , B. Sandhoefer , A. Hansen , and F. Neese , “Natural Triple Excitations in Local Coupled Cluster Calculations With Pair Natural Orbitals,” Journal of Chemical Physics 139, no. 13 (2013): 134101, 10.1063/1.4821834. 24116546

[34] Y. Guo , C. Riplinger , D. G. Liakos , U. Becker , M. Saitow , and F. Neese , “Linear Scaling Perturbative Triples Correction Approximations for Open‐Shell Domain‐Based Local Pair Natural Orbital Coupled Cluster Singles and Doubles Theory [DLPNO‐CCSD(T0/T)],” Journal of Chemical Physics 152, no. 2 (2020): 024116, 10.1063/1.5127550. 31941297

[35] Y. Guo , C. Riplinger , U. Becker , et al., “Communication: An Improved Linear Scaling Perturbative Triples Correction for the Domain Based Local Pair‐Natural Orbital Based Singles and Doubles Coupled Cluster Method [DLPNO‐CCSD(T)],” Journal of Chemical Physics 148, no. 1 (2018): 011101, 10.1063/1.5011798. 29306283

[36] A. Altun , F. Neese , and G. Bistoni , “Extrapolation to the Limit of a Complete Pair Natural Orbital Space in Local Coupled‐Cluster Calculations,” Journal of Chemical Theory and Computation 16, no. 10 (2020): 6142–6149, 10.1021/acs.jctc.0c00344. 32897712 PMC7586325

[37] H. M. Aðalsteinsson and R. Bjornsson , “Ionization Energies of Metallocenes: A Coupled Cluster Study of Cobaltocene,” Physical Chemistry Chemical Physics 25, no. 6 (2023): 4570–4587, 10.1039/D2CP04715B. 36723003

[38] H. Zhai , H. R. Larsson , S. Lee , et al., “Block2: A Comprehensive Open Source Framework to Develop and Apply State‐Of‐The‐Art DMRG Algorithms in Electronic Structure and Beyond,” Journal of Chemical Physics 159, no. 23 (2023): 234801, 10.1063/5.0180424. 38108484

[39] S. Sharma , A. A. Holmes , G. Jeanmairet , A. Alavi , and C. J. Umrigar , “Semistochastic Heat‐Bath Configuration Interaction Method: Selected Configuration Interaction With Semistochastic Perturbation Theory,” Journal of Chemical Theory and Computation 13, no. 4 (2017): 1595–1604, 10.1021/acs.jctc.6b01028. 28263594

[40] A. A. Holmes , N. M. Tubman , and C. J. Umrigar , “Heat‐Bath Configuration Interaction: An Efficient Selected Configuration Interaction Algorithm Inspired by Heat‐Bath Sampling,” Journal of Chemical Theory and Computation 12, no. 8 (2016): 3674–3680, 10.1021/acs.jctc.6b00407. 27428771

[41] R. P. Nagy , “State‐of‐the‐Art Local Correlation Methods Enable Affordable Gold Standard Quantum Chemistry for up to Hundreds of Atoms,” Chemical Science 15, no. 36 (2024): 14556–14584, 10.1039/D4SC04755A. 39246365 PMC11376132

[42] K. Gururangan , J. E. Deustua , J. Shen , and P. Piecuch , “High‐Level Coupled‐Cluster Energetics by Merging Moment Expansions With Selected Configuration Interaction,” Journal of Chemical Physics 155, no. 17 (2021): 174114, 10.1063/5.0064400. 34742204

[43] K. Gururangan and P. Piecuch , “Converging High‐Level Coupled‐Cluster Energetics via Adaptive Selection of Excitation Manifolds Driven by Moment Expansions,” Journal of Chemical Physics 159, no. 8 (2023): 084108, 10.1063/5.0162873. 37610021

[44] B. G. Lippert , J. H. Parrinello , and M. Parrinello , “A Hybrid Gaussian and Plane Wave Density Functional Scheme,” Molecular Physics 92, no. 3 (1997): 477–488, 10.1080/002689797170220.

[45] G. Lippert , J. Hutter , and M. Parrinello , “The Gaussian and Augmented‐Plane‐Wave Density Functional Method for Ab Initio Molecular Dynamics Simulations,” Theoretical Chemistry Accounts 103, no. 2 (1999): 124–140, 10.1007/s002140050523.

[46] S. Grimme , C. Bannwarth , and P. Shushkov , “A Robust and Accurate Tight‐Binding Quantum Chemical Method for Structures, Vibrational Frequencies, and Noncovalent Interactions of Large Molecular Systems Parametrized for All Spd‐Block Elements (Z = 1–86),” Journal of Chemical Theory and Computation 13, no. 5 (2017): 1989–2009, 10.1021/acs.jctc.7b00118. 28418654

[47] T. Froitzheim , M. Müller , A. Hansen , and S. Grimme , “G‐xTB: A General‐Purpose Extended Tight‐Binding Electronic Structure Method for the Elements H to Lr (Z=1–103),” ChemRxiv June 24 (2025), 10.26434/chemrxiv-2025-bjxvt.

[48] V. W. D. Cruzeiro , M. Manathunga , K. M. Merz, Jr. , and A. W. Götz , “Open‐Source Multi‐GPU‐Accelerated QM/MM Simulations With AMBER and QUICK,” Journal of Chemical Information and Modeling 61, no. 5 (2021): 2109–2115, 10.1021/acs.jcim.1c00169. 33913331

[49] F. Bosia , P. Zheng , A. Vaucher , T. Weymuth , P. O. Dral , and M. Reiher , “Ultra‐Fast Semi‐Empirical Quantum Chemistry for High‐Throughput Computational Campaigns With Sparrow,” Journal of Chemical Physics 158, no. 5 (2023): 054118, 10.1063/5.0136404. 36754821

[50] M. Repisky , S. Komorovsky , M. Kadek , et al., “ReSpect: Relativistic Spectroscopy DFT Program Package,” Journal of Chemical Physics 152, no. 18 (2020): 184101, 10.1063/5.0005094. 32414255

[51] L. Genovese , A. Neelov , S. Goedecker , et al., “Daubechies Wavelets as a Basis Set for Density Functional Pseudopotential Calculations,” Journal of Chemical Physics 129, no. 1 (2008): 014109, 10.1063/1.2949547. 18624472

[52] L. E. Ratcliff , W. Dawson , G. Fisicaro , et al., “Flexibilities of Wavelets as a Computational Basis Set for Large‐Scale Electronic Structure Calculations,” Journal of Chemical Physics 152, no. 19 (2020): 194110, 10.1063/5.0004792. 33687268

[53] F. D. Malone , A. Mahajan , J. S. Spencer , and J. Lee , “Ipie: A Python‐Based Auxiliary‐Field Quantum Monte Carlo Program With Flexibility and Efficiency on CPUs and GPUs,” Journal of Chemical Theory and Computation 19, no. 1 (2023): 109–121, 10.1021/acs.jctc.2c00934. 36503227

[54] K. Aidas , C. Angeli , K. L. Bak , et al., “The Dalton Quantum Chemistry Program System,” Wiley Interdisciplinary Reviews: Computational Molecular Science 4, no. 3 (2014): 269–284, 10.1002/wcms.1172. 25309629 PMC4171759

[55] J. M. Turney , A. C. Simmonett , R. M. Parrish , et al., “Psi4: An Open‐Source Ab Initio Electronic Structure Program,” Wiley Interdisciplinary Reviews: Computational Molecular Science 2, no. 4 (2012): 556–565, 10.1002/wcms.93.

[56] R. M. Parrish , L. A. Burns , D. G. A. Smith , et al., “Psi4 1.1: An Open‐Source Electronic Structure Program Emphasizing Automation, Advanced Libraries, and Interoperability,” Journal of Chemical Theory and Computation 13, no. 7 (2017): 3185–3197, 10.1021/acs.jctc.7b00174. 28489372 PMC7495355

[57] S. G. Balasubramani , G. P. Chen , S. Coriani , et al., “TURBOMOLE: Modular Program Suite for Ab Initio Quantum‐Chemical and Condensed‐Matter Simulations,” Journal of Chemical Physics 152, no. 18 (2020): 184107, 10.1063/5.0004635. 32414256 PMC7228783

[58] Y. J. Franzke , C. Holzer , J. H. Andersen , et al., “TURBOMOLE: Today and Tomorrow,” Journal of Chemical Theory and Computation 19, no. 20 (2023): 6859–6890, 10.1021/acs.jctc.3c00347. 37382508 PMC10601488

[59] B. Hourahine , B. Aradi , V. Blum , et al., “DFTB+, a Software Package for Efficient Approximate Density Functional Theory Based Atomistic Simulations,” Journal of Chemical Physics 152, no. 12 (2020): 124101, 10.1063/1.5143190. 32241125

[60] B. Hourahine , M. Berdakin , J. A. Bich , et al., “Recent Developments in DFTB+, a Software Package for Efficient Atomistic Quantum Mechanical Simulations,” Journal of Physical Chemistry. A 129, no. 24 (2025): 5373–5390, 10.1021/acs.jpca.5c01146. 40479742 PMC12186617

[61] E. Caldeweyher , C. Bannwarth , and S. Grimme , “Extension of the D3 Dispersion Coefficient Model,” Journal of Chemical Physics 147, no. 3 (2017): 034112, 10.1063/1.4993215. 28734285

[62] E. Caldeweyher , S. Ehlert , A. Hansen , et al., “A Generally Applicable Atomic‐Charge Dependent London Dispersion Correction,” Journal of Chemical Physics 150, no. 15 (2019): 154122, 10.1063/1.5090222. 31005066

[63] H. Kruse and S. Grimme , “A Geometrical Correction for the Inter‐ and Intra‐Molecular Basis Set Superposition Error in Hartree‐Fock and Density Functional Theory Calculations for Large Systems,” Journal of Chemical Physics 136, no. 15 (2012): 154101, 10.1063/1.3700154. 22519309

[64] P. Eastman , R. Galvelis , R. P. Peláez , et al., “OpenMM 8: Molecular Dynamics Simulation With Machine Learning Potentials,” Journal of Physical Chemistry. B 128, no. 1 (2024): 109–116, 10.1021/acs.jpcb.3c06662. 38154096 PMC10846090

[65] H. M. Senn and W. Thiel , “QM/MM Studies of Enzymes,” Current Opinion in Chemical Biology 11, no. 2 (2007): 182–187, 10.1016/j.cbpa.2007.01.684. 17307018

[66] H. M. Senn and W. Thiel , “QM/MM Methods for Biomolecular Systems,” Angewandte Chemie, International Edition 48, no. 7 (2009): 1198–1229, 10.1002/anie.200802019. 19173328

[67] H. M. Senn and W. Thiel , “QM/MM Methods for Biological Systems,” in Atomistic Approaches in Modern Biology: From Quantum Chemistry to Molecular Simulations, ed. M. Reiher (Springer, 2007), 173–290, 10.1007/128_2006_084.

[68] H. Lin and D. G. Truhlar , “Redistributed Charge and Dipole Schemes for Combined Quantum Mechanical and Molecular Mechanical Calculations,” Journal of Physical Chemistry. A 109, no. 17 (2005): 3991–4004, 10.1021/jp0446332. 16833721

[69] L. W. Chung , W. M. C. Sameera , R. Ramozzi , et al., “The ONIOM Method and Its Applications,” Chemical Reviews 115, no. 12 (2015): 5678–5796, 10.1021/cr5004419. 25853797

[70] T. T. Duignan , “The Potential of Neural Network Potentials,” ACS Physical Chemistry Au 4, no. 3 (2024): 232–241, 10.1021/acsphyschemau.4c00004. 38800721 PMC11117678

[71] J. Behler , “Four Generations of High‐Dimensional Neural Network Potentials,” Chemical Reviews 121, no. 16 (2021): 10037–10072, 10.1021/acs.chemrev.0c00868. 33779150

[72] R. Ramakrishnan , P. O. Dral , M. Rupp , and O. A. von Lilienfeld , “Big Data Meets Quantum Chemistry Approximations: The Δ‐Machine Learning Approach,” Journal of Chemical Theory and Computation 11, no. 5 (2015): 2087–2096, 10.1021/acs.jctc.5b00099. 26574412

[73] J. S. Smith , O. Isayev , and A. E. Roitberg , “ANI‐1: An Extensible Neural Network Potential With DFT Accuracy at Force Field Computational Cost,” Chemical Science 8, no. 4 (2017): 3192–3203, 10.1039/C6SC05720A. 28507695 PMC5414547

[74] X. Gao , F. Ramezanghorbani , O. Isayev , J. S. Smith , and A. E. Roitberg , “TorchANI: A Free and Open Source PyTorch‐Based Deep Learning Implementation of the ANI Neural Network Potentials,” Journal of Chemical Information and Modeling 60, no. 7 (2020): 3408–3415, 10.1021/acs.jcim.0c00451. 32568524

[75] A. Paszke , S. Gross , F. Massa , et al., “PyTorch: An Imperative Style, High‐Performance Deep Learning Library,” In Advances in Neural Information Processing Systems, 32 (2019), 8024–8035.

[76] D. M. Anstine , R. Zubatyuk , and O. Isayev , “AIMNet2: A Neural Network Potential to Meet Your Neutral, Charged, Organic, and Elemental‐Organic Needs,” Chemical Science 16, no. 23 (2025): 10228–10244, 10.1039/D4SC08572H. 40342914 PMC12057637

[77] B. Kalita , R. Zubatyuk , D. M. Anstine , et al., Angewandte Chemie International Edition, 65 (2026), e16763, 10.1002/anie.202516763. 41400153 PMC12851018

[78] S. Maes , F. D. Ceuster , M. V. de Sande , and L. Decin , “MACE: A Machine‐Learning Approach to Chemistry Emulation,” Journal of Open Source Software 10, no. 108 (2025): 7148, 10.21105/joss.07148.

[79] B. M. Wood , M. Dzamba , X. Fu , et al., “UMA: A Family of Universal Models for Atoms,” arXiv June 30 (2025), 10.48550/arXiv.2506.23971.

[80] D. S. Levine , M. Shuaibi , E. W. C. Spotte‐Smith , et al., “The Open Molecules 2025 (OMol25) Dataset, Evaluations, and Models,” arXiv May 13 (2025), 10.48550/arXiv.2505.08762.

[81] P. O. Dral , F. Ge , Y.‐F. Hou , et al., “MLatom 3: A Platform for Machine Learning‐Enhanced Computational Chemistry Simulations and Workflows,” Journal of Chemical Theory and Computation 20, no. 3 (2024): 1193–1213, 10.1021/acs.jctc.3c01203. 38270978 PMC10867807

[82] L.‐P. Wang and C. Song , “Geometry Optimization Made Simple With Translation and Rotation Coordinates,” Journal of Chemical Physics 144, no. 21 (2016): 214108, 10.1063/1.4952956. 27276946

[83] J. Kästner , J. M. Carr , T. W. Keal , W. Thiel , A. Wander , and P. Sherwood , “DL‐FIND: An Open‐Source Geometry Optimizer for Atomistic Simulations,” Journal of Physical Chemistry. A 113, no. 43 (2009): 11856–11865, 10.1021/jp9028968. 19639948

[84] R. S. Billeter , J. A. Turner , and W. Thiel , “Linear Scaling Geometry Optimisation and Transition State Search in Hybrid Delocalised Internal Coordinates,” Physical Chemistry Chemical Physics 2, no. 10 (2000): 2177–2186, 10.1039/A909486E.

[85] A. Banerjee , N. Adams , J. Simons , and R. Shepard , “Search for Stationary Points on Surfaces,” Journal of Physical Chemistry 89, no. 1 (1985): 52–57, 10.1021/j100247a015.

[86] J. N. Harvey , M. Aschi , H. Schwarz , and W. Koch , “The Singlet and Triplet States of Phenyl Cation. A Hybrid Approach for Locating Minimum Energy Crossing Points Between Non‐Interacting Potential Energy Surfaces,” Theoretical Chemistry Accounts: Theory, Computation, and Modeling (Theoretica Chimica Acta) 99, no. 2 (1998): 95–99, 10.1007/s002140050309.

[87] G. Mills , H. Jónsson , and G. K. Schenter , “Reversible Work Transition State Theory: Application to Dissociative Adsorption of Hydrogen,” Surface Science 324, no. 2–3 (1995): 305–337, 10.1016/0039-6028(94)00731-4.

[88] H. Jónsson , G. Mills , and K. W. Jacobsen , “Nudged Elastic Band Method for Finding Minimum Energy Paths of Transitions,” in Classical and Quantum Dynamics in Condensed Phase Simulations (WORLD SCIENTIFIC, 1998), 385–404, 10.1142/9789812839664_0016.

[89] G. Henkelman , B. P. Uberuaga , and H. Jónsson , “A Climbing Image Nudged Elastic Band Method for Finding Saddle Points and Minimum Energy Paths,” Journal of Chemical Physics 113, no. 22 (2000): 9901–9904, 10.1063/1.1329672.

[90] D. C. Liu and J. Nocedal , “On the Limited Memory BFGS Method for Large Scale Optimization,” Mathematical Programming 45, no. 1–3 (1989): 503–528, 10.1007/BF01589116.

[91] S. Smidstrup , A. Pedersen , K. Stokbro , and H. Jónsson , “Improved Initial Guess for Minimum Energy Path Calculations,” Journal of Chemical Physics 140, no. 21 (2014): 214106, 10.1063/1.4878664. 24907989

[92] E. A. Coutsias , C. Seok , and K. A. Dill , “Using Quaternions to Calculate RMSD,” Journal of Computational Chemistry 25, no. 15 (2004): 1849–1857, 10.1002/jcc.20110. 15376254

[93] M. Melander , K. Laasonen , and H. Jónsson , “Removing External Degrees of Freedom From Transition‐State Search Methods Using Quaternions,” Journal of Chemical Theory and Computation 11, no. 3 (2015): 1055–1062, 10.1021/ct501155k. 26579757

[94] V. Ásgeirsson , B. O. Birgisson , R. Bjornsson , et al., “Nudged Elastic Band Method for Molecular Reactions Using Energy‐Weighted Springs Combined With Eigenvector Following,” Journal of Chemical Theory and Computation 17, no. 8 (2021): 4929–4945, 10.1021/acs.jctc.1c00462. 34275279

[95] J. Baker , “An Algorithm for the Location of Transition States,” Journal of Computational Chemistry 7, no. 4 (1986): 385–395, 10.1002/jcc.540070402.

[96] A. Hulm , J. C. B. Dietschreit , and C. Ochsenfeld , “Statistically Optimal Analysis of the Extended‐System Adaptive Biasing Force (eABF) Method,” Journal of Chemical Physics 157, no. 2 (2022): 024110, 10.1063/5.0095554. 35840392

[97] J. Comer , J. C. Gumbart , J. Hénin , T. Lelièvre , A. Pohorille , and C. Chipot , “The Adaptive Biasing Force Method: Everything You Always Wanted to Know but Were Afraid to Ask,” Journal of Physical Chemistry. B 119, no. 3 (2015): 1129–1151, 10.1021/jp506633n. 25247823 PMC4306294

[98] A. Lesage , T. Lelièvre , G. Stoltz , and J. Hénin , “Smoothed Biasing Forces Yield Unbiased Free Energies With the Extended‐System Adaptive Biasing Force Method,” Journal of Physical Chemistry. B 121, no. 15 (2017): 3676–3685, 10.1021/acs.jpcb.6b10055. 27959559 PMC5402294

[99] H. Fu , H. Zhang , H. Chen , X. Shao , C. Chipot , and W. Cai , “Zooming Across the Free‐Energy Landscape: Shaving Barriers, and Flooding Valleys,” Journal of Physical Chemistry Letters 9, no. 16 (2018): 4738–4745, 10.1021/acs.jpclett.8b01994. 30074802

[100] A. Hulm , R. P. Schiller , and C. Ochsenfeld , “Combining Fast Exploration With Accurate Reweighting in the OPES‐eABF Hybrid Sampling Method,” Journal of Chemical Theory and Computation 21, no. 13 (2025): 6434–6445, 10.1021/acs.jctc.5c00395. 40530684 PMC12243082

[101] J. A. Semelak , A. Zeida , N. O. Foglia , and D. A. Estrin , “Minimum Free Energy Pathways of Reactive Processes With Nudged Elastic Bands,” Journal of Chemical Theory and Computation 19, no. 18 (2023): 6273–6293, 10.1021/acs.jctc.3c00366. 37647166

[102] H. Chen , H. Fu , C. Chipot , X. Shao , and W. Cai , “Overcoming Free‐Energy Barriers With a Seamless Combination of a Biasing Force and a Collective Variable‐Independent Boost Potential,” Journal of Chemical Theory and Computation 17, no. 7 (2021): 3886–3894, 10.1021/acs.jctc.1c00103. 34106706

[103] P. Pracht , F. Bohle , and S. Grimme , “Automated Exploration of the Low‐Energy Chemical Space With Fast Quantum Chemical Methods,” Physical Chemistry Chemical Physics 22, no. 14 (2020): 7169–7192, 10.1039/C9CP06869D. 32073075

[104] P. Pracht , S. Grimme , C. Bannwarth , et al., “CREST—A Program for the Exploration of Low‐Energy Molecular Chemical Space,” Journal of Chemical Physics 160, no. 11 (2024): 114110, 10.1063/5.0197592. 38511658

[105] L. Martínez , R. Andrade , E. G. Birgin , and J. M. Martínez , “PACKMOL: A Package for Building Initial Configurations for Molecular Dynamics Simulations,” Journal of Computational Chemistry 30, no. 13 (2009): 2157–2164, 10.1002/jcc.21224. 19229944

[106] G. Schaftenaar and J. H. Noordik , “Molden: A Pre‐ and Post‐Processing Program for Molecular and Electronic Structures*,” Journal of Computer‐Aided Molecular Design 14, no. 2 (2000): 123–134, 10.1023/A:1008193805436. 10721501

[107] T. Lu , “A Comprehensive Electron Wavefunction Analysis Toolbox for Chemists, Multiwfn,” Journal of Chemical Physics 161, no. 8 (2024): 082503, 10.1063/5.0216272. 39189657

[108] E. Posenitskiy , V. G. Chilkuri , A. Ammar , et al., “TREXIO: A File Format and Library for Quantum Chemistry,” Journal of Chemical Physics 158, no. 17 (2023): 174801, 10.1063/5.0148161. 37144717

[109] Y. Pang and R. Bjornsson , “Understanding the Electronic Structure Basis for N2 Binding to FeMoco: A Systematic Quantum Mechanics/Molecular Mechanics Investigation,” Inorganic Chemistry 62, no. 14 (2023): 5357–5375, 10.1021/acs.inorgchem.2c03967. PMC10091479 36988551

[110] Y. Pang and R. Bjornsson , “The E3 State of FeMoco: One Hydride, Two Hydrides or Dihydrogen?,” Physical Chemistry Chemical Physics 25, no. 31 (2023): 21020–21036, 10.1039/D3CP01106B. 37522223

[111] K. Sengupta , J. P. Joyce , L. Decamps , et al., “Investigating the Molybdenum Nitrogenase Mechanistic Cycle Using Spectroelectrochemistry,” Journal of the American Chemical Society 147, no. 2 (2025): 2099–2114, 10.1021/jacs.4c16047. 39746667 PMC11744760

[112] M. A. Martini , K. Bikbaev , Y. Pang , et al., “Binding of Exogenous Cyanide Reveals New Active‐Site States in [FeFe] Hydrogenases,” Chemical Science 14, no. 11 (2023): 2826–2838, 10.1039/D2SC06098A. 36937599 PMC10016341

[113] R. M. Evans , S. E. Beaton , P. R. Macia , et al., “Comprehensive Structural, Infrared Spectroscopic and Kinetic Investigations of the Roles of the Active‐Site Arginine in Bidirectional Hydrogen Activation by the [NiFe]‐Hydrogenase ‘Hyd‐2’ From *Escherichia coli* ,” Chemical Science 14, no. 32 (2023): 8531–8551, 10.1039/D2SC05641K. 37592998 PMC10430524

[114] R. J. Labidi , B. Faivre , P. Carpentier , et al., “Light‐Driven Hydrogen Evolution Reaction Catalyzed by a Molybdenum–Copper Artificial Hydrogenase,” Journal of the American Chemical Society 145, no. 25 (2023): 13640–13649, 10.1021/jacs.3c01350. 37307141

[115] G. Quintil , L. Diebold , G. Fadel , et al., “CO to Isonitrile Substitution in Iron Cyclopentadienone Complexes: A Class of Active Iron Catalysts for Borrowing Hydrogen Strategies,” ACS Catalysis 14, no. 10 (2024): 7795–7805, 10.1021/acscatal.4c01506.

[116] K. M. Byrne , R. Bjornsson , and T. Krämer , “The Diradicaloid Electronic Structure of Dialumenes: A Benchmark Study at the Full‐CI Limit,” Physical Chemistry Chemical Physics 26, no. 48 (2024): 30018–30034, 10.1039/D4CP03005B. 39624956

[117] C. Van Stappen , H. Dai , A. Jose , S. Tian , E. I. Solomon , and Y. Lu , “Primary and Secondary Coordination Sphere Effects on the Structure and Function of S‐Nitrosylating Azurin,” Journal of the American Chemical Society 145, no. 37 (2023): 20610–20623, 10.1021/jacs.3c07399. 37696009 PMC10539042

[118] Q. Lam , C. Van Stappen , Y. Lu , and S. A. Dikanov , “HYSCORE and QM/MM Studies of Second Sphere Variants of the Type 1 Copper Site in Azurin: Influence of Mutations on the Hyperfine Couplings of Remote Nitrogens,” Journal of Physical Chemistry. B 128, no. 14 (2024): 3350–3359, 10.1021/acs.jpcb.3c08194. 38564809

[119] C. Van Stappen , E. Reijerse , S. Chabbra , A. Schnegg , and Y. Lu , “Contrasting Secondary Coordination Sphere Effects on Spin Density Distribution in Red vs. Blue cu Azurin,” JBIC Journal of Biological Inorganic Chemistry 30 (2025): 397–410, 10.1007/s00775-025-02116-x. 40413301

[120] C. Sun , G. Lu , B. Chen , et al., “Direct Asymmetric Synthesis of β‐Branched Aromatic α‐Amino Acids Using Engineered Phenylalanine Ammonia Lyases,” Nature Communications 15, no. 1 (2024): 8264, 10.1038/s41467-024-52613-x. PMC11427684 39327443

[121] P. J. Almhjell , K. E. Johnston , N. J. Porter , et al., “The β‐Subunit of Tryptophan Synthase Is a Latent Tyrosine Synthase,” Nature Chemical Biology 20, no. 8 (2024): 1086–1093, 10.1038/s41589-024-01619-z. 38744987 PMC11288773

[122] B. Li , J. Xiao , Y. Gao , J. Z. H. Zhang , and T. Zhu , “Transition State Searching Accelerated by Neural Network Potential,” Journal of Chemical Information and Modeling 65, no. 5 (2025): 2297–2303, 10.1021/acs.jcim.4c01714. 39977623

[123] G. Greene‐Diniz , C. N. Self , M. Krompiec , et al., “Measuring Correlation and Entanglement Between Molecular Orbitals on a Trapped‐Ion Quantum Computer,” Scientific Reports 15, no. 1 (2025): 28409, 10.1038/s41598-025-04365-x. 40759679 PMC12322021

[124] M. Scheurer , P. Reinholdt , E. R. Kjellgren , J. M. Haugaard Olsen , A. Dreuw , and J. Kongsted , “CPPE: An Open‐Source C++ and Python Library for Polarizable Embedding,” Journal of Chemical Theory and Computation 15, no. 11 (2019): 6154–6163, 10.1021/acs.jctc.9b00758. 31580670

